# Developing strategies and quality control in the Ageing and Brain Working Study in ELSA-Brasil

**DOI:** 10.11606/s1518-8787.2025059006956

**Published:** 2026-01-12

**Authors:** Alessandra Vanessa Lopes Quidim, Raphael Beuttler, Helena Mendes Eloi, Maria Concepción García Otaduy, José Gilvam Araújo Lima Júnior, Murillo Mariotto Teixeira, Carlos Eduardo de Lima Moraes Dardis, Khallil Taverna Chaim, Adriana Bastos Conforto, Claudia da Costa Leite, Carolina de Medeiros Rimkus, Claudia Kimie Suemoto, Angelita Gomes Souza, Itamar Souza Santos, Paulo Andrade Lotufo, Isabela Judith Martins Benseñor, Alessandra Carvalho Goulart

**Affiliations:** I Universidade de São Paulo. Hospital Universitário. Centro de Pesquisa Clínica e Epidemiológica. São Paulo, SP, Brasil; II Universidade de São Paulo. Hospital das Clínicas da Faculdade de Medicina. Instituto e Departamento de Radiologia, Laboratório de Investigação Médica em Radiologia 44. São Paulo, SP, Brasil; III Universidade de São Paulo. Faculdade de Medicina. São Paulo, SP, Brasil; IV Universidade de São Paulo. Faculdade de Saúde Pública. Departamento de Epidemiologia. São Paulo, SP, Brasil

**Keywords:** Data Management, Knowledge Management for Health Research, Management of Science, Technology and Innovation in Health, Manuals and Guides for Research Management

## Abstract

**OBJECTIVE:**

To describe all stages of developing strategies and quality control in the Aging and Brain Working Study linked to the *Estudo Longitudinal de Saúde do Adulto* (ELSA-Brasil – Brazilian Longitudinal Study of Adult Health), a prospective cohort of 15,105 civil servants (aged 35–74) followed up since 2008.

**METHODS:**

Based on the sample of participants alive at the beginning of the sample selection in September 2022 (n = 4,566) from ELSA-Brasil (Centro Investigação São Paulo), a representative sample of 2,165 individuals was calculated according to the following criteria and divided into two subsamples: (1) Sample of participants < 70 years old in Wave 3 (2017–2019) randomized according to cognitive trajectory from Wave 1 to 3, n = 1,670; (2) SuperAgers case-control sample*,* individuals aged ≥ 70 years in Wave 3, based on episodic memory in Wave 3, n = 495 participants (171 cases, 324 controls). The processes implemented to ensure quality control of information prior to data collection were selection of data collection instruments, training and certification of teams, preparation of a manual of definitions and standardized operating procedures, pilot studies, data collection logistics, harmonization of imaging protocols, and creation of an anti-noise device. Given the scope of the study, its multicenter nature, and the diversity of measures involved, this project required the implementation of effective quality assurance and control protocols, ensuring standardization, methodological consistency, and data integrity at all stages.

**RESULTS:**

After implementing the quality assurance and control processes, the final stages related to clinical and imaging data collection, management, and continuous monitoring of field activities were conducted, with weekly reports and statistical monitoring of the sample.

**CONCLUSIONS:**

The adoption of systematic stages of development and quality control was fundamental to ensure the production and reliability of information from brain neuroimaging studies.

## INTRODUCTION

Epidemiological studies characterized using standardized and designed methods that consider the specificities of a population are essential to provide reliable data. The quality of data produced in cohort studies, which are characterized by long-term follow-up, great detail, and verification of collected information, requires rigorous quality control to ensure the excellence of the results obtained^1^.

In this context, the *Estudo Longitudinal de Saúde do Adulto* (ELSA-Brasil – Brazilian Longitudinal Study of Adult Health), a multicenter cohort study that aims to investigate the incidence and risk factors for chronic diseases, particularly cardiovascular and metabolic diseases and diabetes^2,3^, is being conducted in six centers of excellence in research and teaching, involving 15,105 civil servants of both sexes (aged 35 to 74 years), and stands out for its rigorous quality control since its inception (baseline: 2008–2010)^4^.

Fourteen years after the beginning of the main study, with the aging of the cohort, an ancillary study was proposed to assess brain aging with high-resolution neuroimaging (3T and 7T Tesla magnetic resonance imaging). The Aging and Brain Working Study linked to ELSA-Brasil has as its main objective to assess the determinants of the brain aging process, closely related to cognitive and mental health trajectories, which may be associated with chronic conditions and noncommunicable risk factors, such as those of cardiovascular origin and lifestyle habits. In addition, this study aims to investigate successful aging of SuperAgers: individuals over 80 years of age with episodic memory at least as good as average individuals who are two to three decades younger^5^. Considering the multidimensionality of the exposure variables collected since the beginning of the main study, the diversity of the ELSA-Brasil sample in relation to sociodemographic characteristics, such as race, age, gender, and educational level, unlike other cohorts from developed countries that evaluate aging^6^, including studies of SuperAgers^9^, allows for advances in the face of this new reality in a unique and innovative way to gain deeper knowledge in the area. Therefore, with the development and implementation of effective quality assurance and control protocols, standardization, and methodological consistency, ELSA-Brasil ensures greater data integrity at all stages of the long-term study.

The main objective of this article is to describe the developing strategies, quality control and the measures adopted to ensure the quality control of the information generated by the Aging and Brain Working Study at one of the ELSA-Brasil Research Centers in São Paulo.

## METHODS

### Population and Study Design

This is a prospective cohort study to assess the brain aging process in ELSA-Brasil.

Briefly, ELSA-Brasil is a multicenter cohort composed of 15,105 civil servants, aged between 35 and 74 years at baseline, recruited between 2008 and 2010. The main objective of the study is to investigate the incidence and risk factors for chronic diseases, in particular cardiovascular diseases and diabetes^2,3^. As proposed in this ancillary study, ELSA-Brasil, as a pioneering Brazilian long-term cohort, also adopted strategies to ensure the quality control of data collected since baseline^4^.

For the present study, a representative sample of living participants (n = 4,566) from the São Paulo Research Center of ELSA-Brasil^2^ was considered. A sample of 2,165 individuals was calculated based on the following criteria and divided into two subsamples: (1) A sample of participants under 70 years of age in Wave 3 (2017–2019), totaling 1,670 participants, randomized based on the cognitive trajectory from Wave 1 to Wave 3, calculated by the total z-score predicted by regression-based norms from the cognitive battery data (memory + fluency + trail-making test scores) according to age, education, and sex*;* and (2) A sample for the SuperAgers case-control study*,* individuals aged 70 years or older who had late memory scores equal to or higher than the median scores of individuals aged between 46 and 54 years in Wave 3, compared to their sex and age matches, totaling 495 participants (cases = 171, controls = 324).

**Figure 1:**
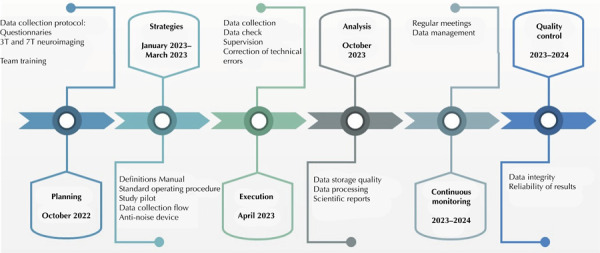
ELSA-Brasil development and quality control stages.

In this aging study, a standardized research protocol was developed for collecting clinical data focusing on cognition, mental health, and high-resolution neuroimaging (3 and 7 Tesla MRI) for an in-depth study of the structural and functional brain aging process, taking into account the harmonization of imaging data with internationally used protocols in other cohorts that included neuroimaging data and that, like ELSA-Brasil, are also dedicated to the study of determinants of brain aging^2,3,6^. The processes implemented to ensure the quality control of information before starting data collection were: the selection of data collection instruments, training and certification of teams, preparation of a manual of definitions and standardized operating procedures (SOPs), pilot studies, data collection flow, harmonization of imaging protocols, and creation of an anti-noise device (Figure 1). All these processes are detailed throughout the text.

Chart 1 presents the logistics and processes developed by the study.

**Chart 1:** Logistics and procedures for conducting brain neuroimaging study in ELSA-Brasil (Aging and Brain Working Study).

.
Development of the research project
Definition and pretesting of the imaging protocol
Selection and pretesting of validated questionnaires and screening scales focusing on cognition, mental health, functionality, and frailty
Manuals defining medical terms and standardized operating procedures
Training and certification of the data collection and scheduling team
Pilots of 3T and 7T MRI imaging exams to verify consistency and reproducibility in a timely manner tolerable by the participant
Pilots to verify data collection flow in the field, including total time per participant (reading and signing documents, completing questionnaires, and changing clothes)
Preliminary data collection
Adaptation of research instruments
Development of a device for fixation and noise reduction for MRI
Protocol for storing images on an external hard drive (backup), in addition to the virtual environment
Protocol for acquiring and archiving clinical data
Data management and periodic reports
Quality control throughout the study

MRI: magnetic resonance imaging; HD: hard disk.

### Research Project Development

The process of creating the standardized research protocol for collecting high-resolution neuroimaging data and assessing structural and functional brain aging took into account the study’s objectives with a focus on aging, prospective design with a two-year follow-up after neuroimaging, population characteristics, including the assessment of participants over 70 years of age, the sample size, and the initial choice of imaging protocol and data collection instruments to be applied for multidimensional assessment of brain aging in men and women.

The research team considered logistical, operational, and financial aspects throughout the process, considering the opinion, understanding, and acceptability of the participants for the adjustments made to the original protocol, both in the pilot phase and during the execution of the study. A team of clinical researchers (epidemiologists, neurologists, geriatricians, and psychiatrists), neuroradiologists, and medical physicists organized themselves into specific committees to define the imaging protocol and select the clinical data collected.

### Imaging Protocol

To standardize the protocol, a sequence of images lasting approximately one hour was obtained for both 3T and 7T MRI. Subsequently, harmonization was performed with international protocols used in other cohorts that evaluated brain aging, following parameters comparable to the European Prevention of Alzheimer’s Dementia Longitudinal Cohort Study (EPAD)^6^. The multiplanar weighted sequences are described in Chart 2. The protocol was performed without the use of paramagnetic contrast (gadolinium), except when an image suspected of active lesion (e.g., tumor, meningioma) was identified. In this case, a new appointment was made to repeat the exam with the use of contrast.

**Chart 2:** Main sequences with respective characteristics of images acquired by magnetic resonance imaging (3T and 7T MRI) in ELSA-Brasil (Aging and Brain Working Study).

Main characteristics	3T magnetic resonance imaging Intera Achieva, PHILIPS Healthcare (Best, Netherlands) with a 32-channel head coil	7T magnetic resonance imaging Magnetom, SIEMENS Healthineers (Erlangen, Germany) with a 32-channel head coil
T1-weighted anatomical image, useful for segmentation of brain structures, assessment of atrophy, enlargement of perivascular spaces, assessment of lacunae and microinfarcts.	**Sagittal 3D T1-weighted fast field echo (FFE):** isotropic resolution of 1 mm	**Sagittal 3D MP2RAGE:** isotropic resolution of 0.75 mm
T2-weighted image with cerebrospinal fluid suppression, useful for visualizing hyperintense lesions, which may be associated with areas of gliosis, intracranial (micro)vascular disease, sequelae infarcts, demyelinating lesions, etc.	**Sagittal 3D Fluid attenuated inversion recovery (FLAIR):** isotropic resolution of 1 mm	**Sagittal 3D fluid attenuated inversion recovery (FLAIR)**: isotropic resolution of 0.75 mm
T2-weighted image, useful for visualizing pathologies with higher water content, such as edema, cysts, or cerebrospinal fluid. Detects hyperintense lesions similar to those detected in FLAIR, with better visualization of lacunae, enlargement of perivascular spaces, and cortical microinfarcts, especially when analyzed in conjunction with other structural sequences.	**Axial 2D T2-weighted Fast Spin-Echo (FSE**): in-plane resolution of 0.9 mm and slice thickness of 3 mm	**Sagittal 3D T2 SPACE:** isotropic resolution of 0.7 mm
Magnetic susceptibility weighted image, useful for visualizing the following factors: - Iron deposition in deep gray matter and other brain compartments: associated with aging and various neurodegenerative diseases, - Hemosiderin deposits: chronic or old bleeding, foci of microhemorrhage associated with amyloid angiopathy and/or intracranial vascular disease; - Calcium deposits: neurocysticercosis, calcified granulomas, and other abnormal calcifications.	**3D Susceptibility-weighted image (SWI):** in-plane resolution of 0.5 mm and slice thickness of 3 mm	**3D Susceptibility-weighted image (SWI):** in-plane resolution of 0.21 mm and slice thickness of 1.5 mm **Axial 3D FFE Multi-gradient echo**: isotropic resolution of 0.7 mm. Sequence performed to obtain quantitative susceptibility mapping (QSM), useful in the complementary evaluation of SWI, for the quantitative assessment of iron deposits and metabolism.
Diffusion-weighted image, useful for detecting acute infarction and for analyzing brain microstructure, tractography, and structural connectivity.	**Axial 2D Diffusion-Weighted (DWI) SE-EPI:** isotropic resolution of 2 mm With two b-values: 0 and 1000 s/mm^2^ 48 gradient directions	**Axial 2D Diffusion-Weighted (DWI) SE-EPI:** isotropic resolution of 2 mm With three b- values: 0.1000, and 2000 s/mm^2^ 30 gradient directions
Dynamic blood oxygenation level dependent (BOLD) contrast-weighted image at rest, useful for assessing brain activity and functional connectivity.	**Axial 2D Resting state-fMRI GRE-EPI:** resolution of 3.3 mm	**Axial 2D Resting state-fMRI GRE-EPI:** Resolution of 2.2 mm
Dynamic arterial spin labeling *(ASL*) image, useful for assessing cerebral perfusion without the need for contrast injection.	**Axial 3D GRASE with ASL:** In-plane resolution of 3.75 mm and slice thickness of 6 mm	

MRI: magnetic resonance imaging.

The first evaluation was a visual analysis of the images obtained in order to assess image quality, presence of artifacts, quantification of white matter lesions (hyperintense lesions), and evaluation of structural brain lesions (Supplementary material: Appendix 1)^a^.

The images were evaluated by two neuroradiologists with at least 10 years of experience in the field. Each exam was interpreted by only one neuroradiologist, who read the qualitative findings described in Appendix 1. In case of disagreement, a consensus was reached between the specialists. Relevant findings with potential impact on the participant’s clinical follow-up, such as intracranial tumors, hemorrhages, infarcts, or other relevant structural lesions, were described in a summary report and delivered to the respective participants. The other qualitative findings were stored in the study database.

### Selection of Research Questionnaires

The instruments used were designed to track symptoms related to the aging process, such as cognitive status (modified telephone interview for cognitive status – TICS-M*)* ^15^, functional assessment (Pfeffer functional activities questionnaire^16^ and frailty questionnaire – FRAIL)^17^, and mental health through the application of the following scales: Dimensions of Anger Reactions Scale (DAR-5)^18^, Positive and Negative Affect Schedule (PANAS)^19^, State-Trait Anxiety Inventory (STAI)^20^, Patient Health Questionnaire (PHQ-9)^13^, Geriatric Depression Scale (GDS-15)^21^, and Distress Scale (K-6)^22^. In addition, we also used instruments on lifestyle habits, including aspects of diet^23^, such as consumption of coffee, processed foods^20^, and alcohol; smoking; physical activity; and sleep^23,24^. The instruments used in the study were validated in Brazil with well-established reproducibility and reliability in the older adults population^25^.

### Manual of Definitions and Standardized Operating Procedures

The definitions manual included clinical diseases and the main urgent and emergency conditions most prevalent in the population, using accessible and standardized language to ensure literacy among the entire research team.

The SOPs developed comprised a detailed compilation of all activities performed, with clear and thorough descriptions related to the measurement of vital signs, head circumference, and guidelines for activating the Blue Code of the Instituto Central do Hospital das Clínicas (ICHC)/Radiology Institute (InRad), including a multidisciplinary rapid response team trained to assist patients in cardiopulmonary arrest (Blue Code) or acute and severe clinical complications (Yellow Code) in non-critical areas of the ICHC and its surroundings (Supplementary material: Appendices 2 to 8)^b^.

### Recruitment of the Research Team

The study began with the identification and recruitment of qualified professionals to compose the study team. After the initial selection, several meetings were held with the main investigator and coordinators to discuss the operational needs of the project. Simultaneously, the acquisition of essential materials began, such as computers, telephones, tablets*,* external clinical and image data storage devices, definition of non-perishable snack kits, ear protectors for use during the exam, chairs with support for right-handed and left-handed individuals to fill out the questionnaires, stationery, and hospital disinfection products.

### Training and Safety Certification for Performing MRI

Specific training was conducted prior to the start of data collection, addressing the necessary precautions for the proper positioning of participants, instructions related to their safety during 3T and 7T MRI exams, and training for administering the safety questionnaire in an MRI environment.

To ensure the safety of the field team while conducting the study in the MRI exam area, they were required to read and sign the good research practices agreement at the Medical Research Laboratory in MRI in Neuroradiology (LIM44) and complete an online certification course on MRI Safety by the team responsible for the neuroimaging sector at InRad-ICHC, FMUSP.

### Training and Certification for Clinical Data Collection

Training was provided to the research team responsible for data collection in the field: (1) a field nurse manager responsible for welcoming participants and providing guidance on clinical and imaging data collection in the field; (2) a public health professional responsible for managing, selecting, and archiving the images acquired; and (3) two nurses assigned to data collection, application of the informed consent form (ICF), and guidance on the correct completion of questionnaires/scales via tablet.

The training and certification of clinical instruments, with a total workload of 9 hours, was carried out by the ELSA-Brasil team responsible for quality control of the São Paulo Center questionnaires, focusing on the application of scales and questionnaires.

In addition to training for the in-person application, interviewers were also certified to apply the same questionnaires by telephone in years 1 and 2 after neuroimaging acquisition. The team was also trained to perform anthropometric measurements (weight, height), head circumference, and casual blood pressure measurement, based on established techniques^3,30,31^.

Diction—including tone of voice and pauses—and the time taken to administer and explain the questionnaires and scales were also standardized^3^. These elements were considered key requirements to ensure that the questions were interpreted uniformly throughout the data collection process. Diction training proved to be fundamental to ensuring accuracy, clarity, and effective communication, contributing to the proper understanding of the questions and the quality of the responses. Controlled tone, in a neutral and well-modulated tone, fostered empathy, creating an environment of trust—especially important in sensitive contexts, such as those involving mental health.

In addition, reading specific words with emphasis and making strategic pauses enabled better processing and understanding of the questions by participants, which contributed to the validity of the responses obtained. The team’s awareness of the importance of respecting the recommended time intervals for applying the instruments, as well as maintaining a neutral posture and diction—without influencing the responses—reinforced the methodological rigor of the study^3^.

Thus, investing in this training was essential to obtain high-quality and reliable data.

On average, the application of the in-person protocol took around 90 minutes (questionnaires, scales, and ICF). A major challenge was training interviewers to guide the self-application of all instruments and scales, except for the TICS-M, in older individuals (mean age of 67 years, ranging from 47 to 90 years at baseline), in particular.

### Training for Case Scheduling

A specialized team, consisting of a nurse and a public health professional, was responsible for scheduling participants. They also participated in practical simulations for inviting participants and administering questionnaires by telephone, using standardized scripts prepared by the project supervisor and field coordinators. Additional training was provided on filling out documentation with sensitive participant data, requesting tests, and sending standardized emails to InRad and participants, including reminder messages sent 48 hours before the test, in order to minimize absences or dropouts.

Two certification and recertification training sessions were held in 2023, before the start of the study, focusing on aspects of scheduling MRI appointments by phone and the specifics involved in the process of obtaining a sequence of quality images. Among these guidelines, the recommendation to maintain a light diet (without the need for prior fasting) and the importance of keeping the head still while the participant is inside the equipment were highlighted.

### Study Pilots

The study team conducted pilot exams on the MRI equipment to optimize logistics and anticipate possible barriers that could hinder the collection of clinical and imaging data. The main objective was to establish a consistent, reproducible, and feasible imaging protocol within a tolerable time frame for study participants.

At the end of the pilot, it was determined that each exam in the 3T MRI machine would take approximately one hour. The reception, informed consent form signing, and questionnaire completion stages took a total of about 3 hours and 30 minutes, in the absence of complications.

On the day of the 7T MRI exam, two brief questionnaires were administered: one on safety, before the procedure, and another after the exam, to assess any discomfort or symptoms reported during imaging. The total estimated time for this stage was, on average, two hours, also in the absence of complications.

### Preliminary Data Collection and Instrument Adaptation

After defining the care flows, the study team evaluated the selected instruments for comprehensibility after including the first 20 participants. Based on this stage, minor modifications and adaptations were made without compromising the validity and content of the instruments. The implementation team was properly trained in the correct use of the instruments, ensuring consistency in data collection.

This process ensured that the research instruments were appropriate, validated, and reliable for the target population and compatible with the study objectives.

### Development of a Head Stabilization and Noise Reduction Device for MRI

The pilot study, conducted with five volunteer participants (aged between 40 and 65), identified that the main complaint during MRI exams was the noise generated by the machine, especially in 7T MRI exams, even with the use of standard hearing protectors. This complaint was concerning, as it could lead to unwanted head movements, compromising the quality of the image obtained.

In response to this problem, the research team—composed of a medical physicist and a technologist specializing in biomechanics and assistive technology—began developing a prototype head stabilizer and noise reducer in 2023, inspired by models already marketed in Europe and the United States, which in Brazil are only available through high-cost imports. The new prototype provides comfortable head stabilization, ensuring better image quality during exams.

The team evaluated the original material of the internationally marketed device and, based on that, began manufacturing the prototype, which consists of two PVC bags for each ear, connected to two rubber cannulas and three resin valves. These valves were produced by 3D printing and allow the bags to be inflated, which are sealed by a sealer. The system provides head stabilization and noise reduction, significantly improving the acoustic sensation during the exam.

**Figure 2:**
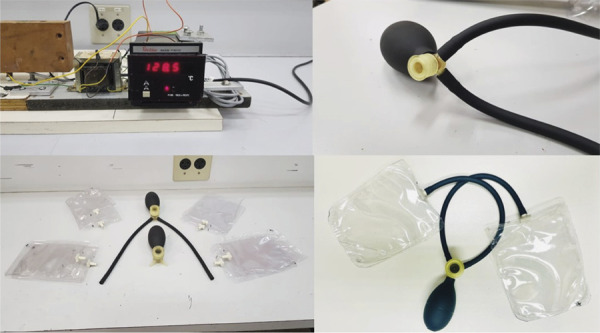
Prototype of the noise protector produced by the research team during the ELSA-Brasil study.

The prototype was tested with research participants, and all materials used were considered safe for use in magnetic field environments. Figure 2 shows the sealer developed to seal the PVC bags, as well as the other materials used (rubber connectors and resin valves). The results of these tests contributed significantly to improving the participants’ experience and the quality of the images obtained in MRI exams.

### Image Data Storage Protocol

A protocol was also developed for storing images on an external hard drive, in addition to the InRad and PISA cloud platforms. After the exams were performed, the images were extracted from the InRad (3T MRI) and PISA (7T MRI) virtual systems and transferred to external storage on optical media*—*compact disc/digital versatile disc (CD/DVD)—properly identified with the participant’s name, ID_ELSA, date and time of the exam, date of birth, RGHC, identification number in the InRad virtual system, name of the protocol, and type of exam performed.

In accordance with the General Personal Data Protection Law, all access to participants’ images was password protected and restricted exclusively to the operational team and the researchers in charge.

## RESULTS

After implementing the quality assurance and control processes prior to data collection, the final stages related to clinical data collection, management, and continuous monitoring of field activities were conducted, with the preparation of weekly reports and statistical monitoring of the sample.

Clinical data collection was performed using digital forms (Google Forms®), self-completed by participants on tablets accessed with individual passwords provided by the research team, under real-time supervision by ELSA-Brasil interviewers. Upon completion, the data were automatically stored in structured spreadsheets, managed by interviewers and field supervisors. This strategy ensured the integrity, confidentiality, and traceability of the information, optimizing its use in subsequent stages of the research. The data were kept in a secure environment (Google Drive®), with access restricted to the field team and main investigators—the latter with read-only permission. Any inconsistencies in the questionnaires were verified and corrected exclusively by the data management team, with daily supervision by two coordinators. The monitoring of the ELSA-Brasil Aging and Brain Working Study team was intensive and structured, involving daily supervision by two coordinators. This monitoring covered the entire data collection process, including documentation management, forecasting and provision of human, food, and material resources, monitoring of databases and statistics, control of reports, as well as image management and storage. Periodic observations were aimed at identifying the need for adjustments to the care flowchart, protocol deviations, and aspects of data collection quality. Care flowcharts, checklists, and standardized scripts were used for the application of the ICF and questionnaires.

The interviewers checked the answers filled out by the participants during the application of the questionnaires in order to identify inconsistencies and/or errors, enabling immediate correction in the presence of the participant. Weekly reports and sample statistics were produced by field supervisors, ensuring compliance with recruitment goals and control of participant adherence.

Finally, a feedback mechanism was created for participants to evaluate the quality of service and the responsiveness of the team. Opinions, suggestions, and criticisms were received through a messaging app.

## DISCUSSION

The quantification of biological aging, especially in the context of brain aging, is essential for understanding the mechanisms underlying cognitive decline and for identifying risk and protective factors for the brain in aging over time.

In the Brazilian context, ELSA-Brasil stands out for its robust methodology, which includes comprehensive quality assurance strategies, such as constant equipment calibration, systematic audits, and ongoing training of teams involved in data collection and analysis^2,3^. The recent integration of neuropsychological and neuroimaging assessments expands ELSA-Brasil’s potential to investigate not only typical aging (cognitive decline), but also the aging of *SuperAgers,* who have cognitive performance equal to or superior to that of much younger people, especially in memory.

Complementarily, the Rotterdam Study^6^ is a consolidated reference in the international scenario, with more than three decades of rigorous and standardized follow-up of a population cohort. Maintaining methodological consistency over time, with uniform clinical and laboratory protocols, as well as centralized data control systems, has enabled the identification of early biomarkers and trajectories of healthy and pathological aging.

Large longitudinal studies, such as ELSA-Brasil^2,3^and the Rotterdam Study^6^, have been fundamental in this scenario, as they have enabled the analysis of different aging trajectories based on the systematic collection of clinical, cognitive, and neuroimaging data. The reliability of these analyses depends directly on the rigorous implementation of standardized protocols and quality control, ensuring the validity and comparability of results between different phases of the study and between different populations.

Other studies conducted in developed countries such as the United States, Spain, and Australia that evaluated samples of *SuperAgers* shared a concern with cognitive preservation in aging, with careful sample selection and strict control of methodological and data quality, ensuring that the findings are reliable and useful for understanding the factors that protect the brain in healthy aging^9^.

Thus, the integration of the findings from these different studies^2,3,6^reinforces the importance of quality control and standardization in measuring biological aging, contributing to the development of effective strategies to promote healthy brain aging and prevent large-scale cognitive decline.

## CONCLUSION

The implementation of systematic development and quality control steps was essential to ensure the production and reliability of data in high-resolution brain neuroimaging studies (3T and 7T) aimed at investigating functional and structural aging, considering the methodological complexity and intrinsic sensitivity of the information obtained in the Aging and Brain Working Study in ELSA-Brasil.

## Supplementary material

Appendix 1

## Footnotes

a Supplementary material available from: https://figshare.com/s/da0e932b6f1f0b36fae5

b Supplementary material available at: https://figshare.com/s/da0e932b6f1f0b36fae5
