# Supplementary material for: Developing strategies and quality control in the Ageing and Brain Working Study in ELSA-Brasil
Source: Rev Saude Publica. 2026 Jan 12;59:e54. doi: 10.11606/s1518-8787.2025059006956 (PMC12795433; doi:10.11606/s1518-8787.2025059006956)
Supplement: Appendix 1 [file 1518-8787-rsp-59-e54-suppl01.pdf]

Anexo 1. Análise visual das imagens cerebrais captadas pela Ressonância Magnética (RM) do Crânio RM 3T - Protocolo ELSA-Cérebro

1. Nome:

2. Data de Nascimento:

3. ID ELSA:

4. ID PACS

5. Data do Exame

6. Qualidade de imagem

   Marcar apenas uma opção

   - ⬭ Boa sem artefatos (*Pular para a pergunta 9*)
   - ⬭ Boa com poucos artefatos
   - ⬭ Ruim passível de laudo
   - ⬭ Ruim não passível de laudo

7. Tipo de artefato

   Marcar apenas uma opção

   - ⬭ Movimento (*Pular para a pergunta 9*)
   - ⬭ Suscetibilidade magnética
   - ⬭ Outros problemas técnicos
   - ⬭ NA

8. Observações

Sistema Ventricular

9. Dilatação Ventricular

Marcar apenas uma opção

- Ausente (*Pular para a pergunta 11*)
- Leve
- Moderada
- Acentuada

Sistema Ventricular

10. Dilatação Ventricular

Marcar apenas uma opção

- Hipertensiva
- Não Hipertensiva

Cisternas e Sulcos

11. Cisternas e Sulcos (escala de atrofia cortical Pasquier - *Global Cortical Atrophy*)

Marcar apenas uma opção.

- GCA 0 (ausência de alargamento), *pular para a pergunta 17*
- GCA 1 (discreto alargamento de sulcos)
- GCA 2 (moderado alargamento dos sulcos com leve perda de volume dos giros)
- GCA 3 (acentuado alargamento dos sulcos em aspecto de lâmina de faca)

Cisternas e Sulcos

12. Localização do alargamento de sulcos / atrofia:

Marcar apenas uma opção.

- Difuso, sem predomínio regional (*Pular para a pergunta 17*)
- Com predomínio regional

Cisternas e Sulcos

13. Localização do Alargamento:

Marcar apenas uma opção.

- Frontal (*Pular para a pergunta 17*)
- Temporal (*Pular para a pergunta 17*)
- Parietal (*Pular para a pergunta 17*)
- Occipital (*Pular para a pergunta 17*)
- Cerebelar (*Pular para a pergunta 17*)
- Tronco encefálica (*Pular para a pergunta 17*)

14. Característica do alargamento de sulcos:

Marcar apenas uma opção.

- Simétrico
- Direita
- Esquerda

Atrofia Cortical Posterior (parietal) - Escala de Koedam

15. Escala de atrofia Cortical Posterior (Escala de Koedam) - DIREITA

Marcar apenas uma opção.

- 0
- 1
- 2
- 3

16. Escala de atrofia Cortical Posterior (Escala de Koedam) - ESQUERDA

Marcar apenas uma opção.

- 0
- 1
- 2
- 3

Apagamento dos sulcos

17. Apagamento dos sulcos

Marcar apenas uma opção.

- Não (*Pular para a pergunta 20*)
- Sim

Apagamento dos Sulcos

18. Apagamento dos Sulcos

Marcar apenas uma opção.

- Difuso (*Pular para a pergunta 20*)
- Localizado

Apagamento dos Sulcos

19. Se localizado, topografia

Hipocampos

20. MTA - Escala de Scheltens (DIREITA)

Marcar apenas uma opção.

- 0
- 1
- 2
- 3
- 4

21. MTA - Escala de Scheltens (ESQUERDA)

Marcar apenas uma opção.

- 0
- 1
- 2
- 3
- 4

Córtex Entorrinal

22. Escala do Córtex entorrinal - ERICA (DIREITA)

Marcar apenas uma opção.

- 0
- 1
- 2
- 3

23. Escala do Córtex entorrinal - ERICA (ESQUERDA) *

Marcar apenas uma opção.

- 0
- 1
- 2
- 3

Tronco encefálico

24.Atrofia da ponte

Marcar apenas uma opção.

- Não
- Sim

Parênquima Encefálico

25. Parênquima Encefálico
Lesões arredondadas ou amorfas com hipersinal em T2/FLAIR (positivo se > 3)

Marcar apenas uma opção.

- Não (*Pular para a pergunta 29*)
- Sim

Parênquima Encefálico

26. Parênquima Encefálico

Marque todas que se aplicam.

- Supratentorial
- Infratentorial

Escala de Fazekas

27. Fazekas Periventricular

Marcar apenas uma opção.

- 0
- 1 (se > de 3 focos)
- 2 (confluente inicial)
- 3 (confluente avançado)

28. Fazekas Substância Branca Subcortical

Marcar apenas uma opção.

- 0
- 1
- 2
- 3

Lacunas

29. Lacunas

Marcar apenas uma opção.

- ⬭ Não (*Pular para a pergunta 32*)
- ⬭ Sim

Lacunas

30. Lacunas

Marcar apenas uma opção.

- ⬭ 1
- ⬭ 2 a 5
- ⬭ 6 a 10
- ⬭ >10

31. Lacunas nos talamos

Marcar apenas uma opção.

- ⬭ Não
- ⬭ Unilateral
- ⬭ Bilateral

Infartos Corticais (ISQUÊMICOS)

32. Infartos Corticais *

Marcar apenas uma opção.

- Não (*Pular para a pergunta 35*)
- Sim

Infartos Corticais

33. Infartos Corticais

Marque todas que se aplicam.

- Focal - 1 a 2 giros/sulcos
- Multifocais (focal em múltiplas localizações)
- Lobar único (1 área de encefalomalácea maior do que 1/3 de um território arterial)
- Lobares múltiplos (mais de 1 área de encefalomalácea maior do que 1/3 de um território arterial)
- Cerebelo Focal
- Cerebelo Multifocal
- Cerebelo hemisférico
- Cerebelo bi hemisférico
- Outro

34. Localização da(s) lesões:

Lesões com restrição à difusão

35. Lesões com restrição à difusão *

Marcar apenas uma opção.

- Não (*Pular para a pergunta 37*)
- Sim

Lesões com restrição à difusão

36. Localização de lesões com restrição

Microhemorragia / Calcificação

37. Microhemorragia / Calcificação *

Marcar apenas uma opção.

- Não (*Pular para a pergunta 39*)
- Sim

Localização de microhemorragia / Calcificação

38. Localização de microhemorragia / Calcificação

Marque todas que se aplicam.

- [ ] Substância cinzenta profunda
- [ ] Hemisférica
- [ ] Tronco
- [ ] Cerebelo
- [ ] Outro

Hematoma intraparenquimatoso

39. Hematoma intraparenquimatoso *

Marcar apenas uma opção.

( ) Não (*Pular para a pergunta 41*)
( ) Sim

Hematoma intraparenquimatoso

40. Localização do hematoma intraparenquimatoso

Hemorragia subaracnóide (HSA) aguda/subaguda precoce

41. HSA aguda/subaguda precoce *

Marcar apenas uma opção.

( ) Não (Pular para a pergunta 43)
( ) Sim

Hemorragia subaracnóide (HSA) aguda/subaguda precoce

42. Localização da HSA aguda/subaguda precoce

Hemorragia subaracnóide (HSA) tardia - Siderose Superficial

43. HSA tardia - Siderose Superficial *

Marcar apenas uma opção.

( ) Não (*Pular para a pergunta 45*)
( ) Sim

Hemorragia subaracnóide (HSA) tardia - Siderose Superficial

44. Localização da HSA tardia - Siderose Superficial

Espaços perivasculares

45. Alargamento de espaços de Virchow-Robin *

Marcar apenas uma opção.

- Não (menos do que 10), *Pular para a pergunta 47*
- Sim (mais do que 10)

Alargamentos perivasculares

46. Localização do(s) alargamento(s)

Marque todas que se aplicam.

- Subcortical
- Núcleos da base
- Tálamos
- Hipocampo
- Infratentorial
- Outros

Demais perguntas

47. Outras lesões parenquimatosas (descrever):

48. Lesões extra-axiais (descrever):

49. Observações Livres

RM 7T - Protocolo ELSA Cérebro

1. Nome:

2. ID ELSA:

3. Data do Exame

4. Qualidade de imagem

   Marcar apenas uma opção.

   - Boa sem artefatos Pular para a pergunta 9
   - Boa com poucos artefatos
   - Ruim passível de laudo
   - Ruim não passível de laudo

5. Tipo de artefato

   Marcar apenas uma opção.

   - Movimento (*Pular para a pergunta 9*)
   - Suscetibilidade magnética
   - Outros problemas técnicos
   - N.A

6. Observações

Lacunas

7. Lacunas
   Marcar apenas uma opção.

   - Não (*Pular para a pergunta 10*)
   - Sim

Lacunas

8. Lacunas
Marcar apenas uma opção.

- 1
- 2 a 5
- 6 a 10
- >10

9. Lacunas nos talamos
Marcar apenas uma opção.

- Não
- Unilateral
- Bilateral

Microhemorragia / Calcificação

10. Microhemorragia / Calcificação *

- Não (*Pular para a pergunta 12*)
- Sim

Localização de microhemorragia / Calcificação

11. Localização de microhemorragia / Calcificação

Marque todas que se aplicam.

- [ ] Substância cinzenta profunda
- [ ] Hemisférica
- [ ] Tronco
- [ ] Cerebelo

Espaços perivasculares

12. Alargamento de espaços de Virchow-Robin *
Marcar apenas uma opção.

- [ ] Não (menos do que 10), *Pular para a pergunta 14*
- [ ] Sim (mais do que 10)

Alargamentos perivasculares

13. Localização do(s) alargamento(s)

Marque todas que se aplicam.

- [ ] Subcortical
- [ ] Núcleos da base
- [ ] Tálamos
- [ ] Hipocampo
- [ ] Infratentorial
- [ ] État criblé ou outros

Outras alterações não visualizadas na RM 3T:

14. Observações

Anexo 2. Manual de Definições e Condutas das principais urgências e emergências clínicas para atendimento aos participantes do Estudo ELSA-Brasil

1) **Obstrução de Vias Aéreas por Corpos Estranhos (OVACE):** obstrução de vias aéreas por corpos estranhos. A obstrução pode apresentar oclusão parcial ou total das vias respiratórias, que, consequentemente, pode comprometer o ciclo respiratório do indivíduo e levá-lo a óbito.
Sintomas: dificuldade respiratória associada à tosse, vômito, respiração ruidosa, perda da voz, agitação e ansiedade, podendo evoluir para asfixia e rebaixamento do nível de consciência.
2) **Convulsões (agitações psicomotoras):** É uma contração violenta, ou série de contrações dos músculos voluntários, com ou sem perda de consciência.
Sinais e sintomas: inconsciência, queda desamparada, onde a vítima é incapaz de fazer qualquer esforço para evitar danos físicos a si própria, olhar vago, fixo e/ou revirar dos olhos, suor, midríase (pupila dilatada), lábios cianóticos, espumar pela boca, morder a língua e/ou lábios, corpo rígido e contração do rosto, palidez intenso, movimentos involuntários e desordenados, perda de urina e/ou fezes (relaxamento esfincteriano). Geralmente os movimentos incontroláveis duram de 2 a 4 minutos, tornando-se, então, menos violentos e o acidentado vai se recuperando gradativamente. Estes acessos podem variar na sua gravidade e duração. Depois da recuperação da convulsão há perda da memória, que se recupera mais tarde.
3) **Hiperglicemia:** Taxas elevadas de glicose plasmática. Na presença de sintomas inequívocos de hiperglicemia e teste de glicemia capilar ao acaso >200mg/dl.
Sinais e sintomas: poliúria, polidipsia, perda de peso, polifagia e visão turva ou por complicações agudas que podem levar a risco de vida, como a cetoacidose diabética e a síndrome hiperosmolar hiperglicêmica não cetótica.
4) **Hipoglicemia:** Diminuição da concentração de glicose no sangue para valores <70mg/dL (indivíduos diabéticos) e <50mg/dL (indivíduos não diabéticos).
Sintomas neuroautonômicos (causados pela resposta autonômica): falta de estabilidade, tremor, ansiedade, nervosismo, palpitações, sudorese, pele pegajosa, boca seca, fome, palidez, dilatação da pupila.
Sintomas neuroglicopênicos (causados pela concentração reduzida de glicose no SNC): sensações estranhas (calor, formigamentos), irritabilidade, confusão mental, dificuldade de raciocínio, dificuldade de fala, visão borrada, ataxia, parestesias, dor de cabeça, confusão mental, convulsão, coma e morte.

5) **Infarto do miocárdio:** Necrose do músculo cardíaco após isquemia por oclusão arterial coronariana aguda, ou seja, é um quadro clínico consequente à deficiência de fluxo sanguíneo para uma dada região do músculo cardíaco (miocárdio), cujas células sofrem necrose devido à falta de aporte de oxigênio.
Sinais e sintomas: dor torácica angustiante e/ou insuportável na região precordial (subesternal), retroesternal e face anterior do tórax, compressão no peito e angústia, dor não diminui com repouso, possível Irradiação no sentido da mandíbula e membros superiores, particularmente do membro superior esquerdo, eventualmente para o estômago (epigástrio), pode apresentar arritmia, palpitação, vertigem, desmaio, sudorese profusa, palidez, náusea, vômitos, diarreia, dispneia, tosse com expectoração rósea e parada cardiorrespiratória.
6) **Taquisfigmia:** frequência de pulso acima de valores de normalidade.
Sinais: frequência acima de 100bpm em indivíduo adulto.
7) **Dessaturação:** é uma condição resultante da queda da taxa de oxigênio sanguíneo para níveis abaixo do normal, sendo caracterizada por uma saturação de O2 menor que 95% em ar ambiente, considerando uma amostra de sangue de indivíduo saudável.
Sinais e sintomas: cianose periférica, cianose central e/ou dispneia
8) **Hipotensão arterial:** ocorre quando a pressão arterial cai a ponto de provocar sintomas como tonturas e desmaios. Uma pressão arterial muito baixa pode causar danos a órgãos, um processo chamado choque.
Sinais e sintomas: vertigem ou sensação de desmaio iminente, desmaio, falta de ar ou dor torácica e taquicardia
9) **Hipertensão arterial sistêmica, crise hipertensiva, urgência e emergência hipertensiva:** Sua evolução é eventualmente marcada por episódio de elevação súbita e acentuada da pressão arterial, representando uma séria e grave ameaça à vida.
Urgência: caracterizada por elevações da PA, sem lesões em LOA (lesão em órgão-alvo) e sem risco de morte iminente.
Emergência: apresenta elevação acentuada da PA associada à LOA e risco imediato de morte, podendo se manifestar com evento cardiovascular, cerebrovascular, renal, entre outros.

Sinais e sintomas: encefalopatia, cefaleia intensa, geralmente posterior e na nuca, falta de ar, sensação dos batimentos cardíacos (palpitação), ansiedade, nervosismo, perturbações neurológicas, tontura e instabilidade, zumbido, escotomas cintilantes (visão de pequenos objetos brilhantes), náusea e vômito podem estar presentes.

10) **Mal-estar:** é uma sensação física desagradável, bem como a uma inquietação indefinida
11) **Rebaixamento de nível de consciência**
    Sinais e sintomas: sonolência, letargia, obnubilação, torpor e coma
12) **Parada cardiorrespiratória (PCR):** consiste em uma emergência cardiovascular multifatorial caracterizada pela interrupção súbita da função mecânica ventricular e respiratória, na ausência de consciência, mas com viabilidade cerebral e biológica.
    Sinais e sintomas: dores fortes no peito, que irradiam para as costas ou abdômen, cefaleia intensa, disartria, dispnéia, formigamento no braço esquerdo e palpitações fortes.

13) **Trauma cranioencefálico:** é qualquer lesão decorrente de um trauma que tenha como consequência fratura ou laceração do couro cabeludo, comprometimento funcional e cognitivo resultando em alterações cerebrais, momentâneas ou permanentes.
    Sinais e sintomas: desmaio, perda de memória, dor de cabeça intensa, dificuldade para enxergar ou perda da visão, confusão mental, vômitos, fala alterada, perda de equilíbrio, sangramentos graves na cabeça ou rosto, saída de sangue ou de líquido transparente pelo nariz e ouvidos, olho roxo, perda de sensibilidade em alguma parte do corpo e sonolência excessiva.
14) **Sudorese:** é o termo técnico utilizado para designar o suor.
15) **Cefaleia:** é o termo técnico utilizado para designar a dor de cabeça.
16) **Náuseas e/ou vômitos:** é uma sensação de desconforto que afeta a porção superior do abdômen, mais ou menos na região da boca do estômago. O vômito ou êmese é um ato involuntário, que consiste na expulsão peroral forçada do conteúdo gástrico, provocada por contração enérgica dos músculos abdominais.
17) **Queimaduras:** é toda lesão provocada pelo contato direto com alguma fonte de calor ou frio, produtos químicos, corrente elétrica, radiação, ou mesmo alguns animais e plantas (como larvas, água-viva, urtiga), entre outros.
    Sinais e sintomas:
    - **1º grau:** atingem as camadas superficiais da pele. Apresentam vermelhidão, inchaço e dor local suportável, sem a formação de bolhas;
    - **2º grau:** atingem as camadas mais profundas da pele. Apresentam bolhas, pele avermelhada, manchada ou com coloração variável, dor, inchaço, desprendimento de camadas da pele e possível estado de choque.
    - **3º grau:** atingem todas as camadas da pele e podem chegar aos ossos. Apresentam pouca ou nenhuma dor e a pele branca ou carbonizada.
18) **Desmaio:** É a perda súbita, temporária e repentina da consciência, devido à diminuição de sangue e oxigênio no cérebro.
    Sinais e sintomas: fraqueza, suor frio abundante, náusea ou ânsia de vômito, palidez intensa, pulso fraco, pressão arterial baixa, respiração lenta, extremidades frias, tontura, escurecimento da visão, perda da consciência e queda.
19) **Acidente Vascular Cerebral (AVC):** acontece quando vasos que levam sangue ao cérebro entopem ou se rompem, provocando a paralisia da área cerebral que ficou sem circulação sanguínea.
    Sinais e sintomas: fraqueza ou formigamento na face, no braço ou na perna, especialmente em um lado do corpo, confusão mental, alteração da fala ou compreensão, alteração na visão, alteração do equilíbrio, coordenação, tontura ou alteração no andar, dor de cabeça súbita, intensa, sem causa aparente.

CONDUTAS GERAIS

1. Checar sinais vitais, registrar e comunicar o biomédico, enfermagem do setor e o médico, se necessário.
2. Levar o participante para dentro da ZONA 2, após pedir autorização para equipe do setor, e deixá-lo confortável e seguro em poltrona ou maca, a depender da situação.
2. Avisar também os coordenadores do estudo por aplicativo de mesagem ou e-mail sobre qualquer intercorrência.

OBSERVAÇÕES

1. Em caso de intercorrências, o participante deve ser atendido no setor, seguindo o fluxo setor, conforme POP.

2. O participante não deve sair do setor sem ter avaliação médica documentada (alta ou transferência para o PSHC conforme quadro clínico).

Referências

1. Nery M. Hipoglicemia como fator complicador no tratamento do diabetes melito tipo 1. Arq Bras Endocrinol Metab [Internet]. 2008 Mar;52(2):288-98. doi: https://doi.org/10.1590/S0004-27302008000200016.
2. Bortolotto LA, Silveira JVd, José F, Martin V. Crises hipertensivas: definindo a gravidade e o tratamento. Rev Soc Cardiol Estado de São Paulo [Internet]. 2018 [cited 2023 Mar 29]. Disponível em: http://dx.doi.org/10.29381/0103-8559/20182803254-9. Disponível em: https://socesp.org.br/revista/assets/upload/revista/5579307671539114883pdfenCRISES%20HIPERTENSIVAS%20-%20DEFININDO%20A%20GRAVIDADE_REVISTA%20SOCESP%20V28%20N3.pdf.
3. Silva FL da, Galindo Neto NM, Sá GG de M, França MS de, Oliveira PMP de, Grimaldi MRM. Tecnologias para educação em saúde sobre obstrução das vias aéreas por corpo estranho: revisão integrativa. Rev Esc Enferm USP [Internet]. 2021;55:e03778. doi: https://doi.org/10.1590/S1980-220X2020035103778.
4. Cobas R, Rodacki M, Giacaglia L, Calliari L, Noronha R, Valerio C, Custódio J, Santos R, Zajdenverg L, Gabbay G, Bertoluci M. Diagnóstico do diabetes e rastreamento do diabetes tipo 2. Diretriz Oficial da Sociedade Brasileira de Diabetes (2022). doi: 10.29327/557753.2022-2.
5. Takahashi EIU, Chiarello MdL, Miyadahira AMK, Araújo CP de, Nakamae DD, Valente MA, et al. VÔMITO E HEMATÊMESE: aspectos gerais e conduta de enfermagem. Rev Esc Enferm USP [Internet]. 1980 Dec;14(3):219-27. Disponível em: https://doi.org/10.1590/0080-6234198001400300219.
6. Gross JL, Silveiro SP, Camargo JL, Reichelt AJ, Azevedo MJ de. Diabetes Melito: Diagnóstico, Classificação e Avaliação do Controle Glicêmico. Arq Bras Endocrinol Metab [Internet]. 2002 Feb;46(1):16-26. Disponível em: https://doi.org/10.1590/S0004-27302002000100004.
7. Lopes FJ, Ribeiro JB, Stavale R, Bolzan DW, Guizilini S, Lopes RSM. Desafios no manejo da parada cardiorrespiratória durante a pandemia da COVID-19: um estudo de reflexão. Esc Anna Nery [Internet]. 2020;24(spe):e20200296. Disponível em: https://doi.org/10.1590/2177-9465-EAN-2020-0296.
8. Rede D'Or São Luiz. Náusea [Internet]. Rede D'Or São Luiz; c2023 [atualizado em 2023]. Disponível em: https://www.rededorsaoluiz.com.br/doencas/nausea.
9. Ministério da Saúde. Queimaduras [Internet]. Brasília (DF): Ministério da Saúde; 2023 [atualizado em 23 mar 2023]. Disponível em: https://bvsms.saude.gov.br/queimaduras/#:~:text=Queimadura%20%C3%A9%20toda%20les%C3%A3o%20provocada,%2C%20urtiga)%2C%20entre%20outros.
10. Dicio. Mal-estar, mal estar, mau-estar ou mau estar? [Internet]. c2023 [atualizado em 14 mar 2023]. Disponível em: https://www.dicio.com.br/mal-estar-mal-estar-mau-estar-ou-mau-estar/.

Anexo 3. Manual de definições das principais doenças clínicas do Estudo ELSA-Brasil

Esclerose Múltipla: é uma doença inflamatória crônica, provavelmente autoimune, causada por fatores genéticos ou ambientais. Dessa forma, o sistema imunológico começa a agredir a camada de gordura que envolve as fibras nervosas (bainha de mielina) comprometendo a função do sistema nervoso (cérebro e medula) ligada a comunicação dos neurônios para o resto do corpo. Sinais e sintomas: fadiga, distúrbios visuais, rigidez, fraqueza muscular, desequilíbrio, alterações sensoriais, dor, disfunção da bexiga e/ou do intestino, disfunção sexual, dificuldade para articular a fala, dificuldade para engolir, alterações emocionais e alterações cognitivas.

E.L.A (Esclerose Lateral Amiotrófica): é uma doença que afeta o sistema nervoso de forma degenerativa e progressiva gerando uma paralisia motora irreversível. Não há cura e os portadores da doença sofrem paralisia gradual e morte precoce como resultado da perda de capacidades importantes, como falar, movimentar, engolir e respirar. Sinais e sintomas: Perda gradual de força e coordenação muscular, incapacidade de realizar tarefas rotineiras, dificuldades para respirar e engolir, engasgar com facilidade, gagueira, cabeça caída, cãibras musculares, problemas de dicção (arrastando as palavras), alterações da voz, rouquidão e perda de peso.

Epilepsia: é uma alteração temporária e reversível do funcionamento do cérebro. Durante alguns segundos ou minutos, uma parte do cérebro emite sinais incorretos, que podem ficar restritos a esse local ou se espalhar, não é causada por febre, drogas ou distúrbios metabólicos. Sinais e sintomas: contrações musculares em todo o corpo, mordedura da língua, salivação intensa, respiração ofegante.

Demência: é o declínio geral das habilidades mentais, como memória, linguagem e raciocínio que persiste por toda a vida e pode interferir nas atividades do cotidiano e seus relacionamentos. Apesar de ser mais frequente em idosos, demência não é uma consequência normal do envelhecimento. Sinais e sintomas: perda gradual e progressiva da memória, confusão mental, perda da capacidade de resolver problemas, comportamento agitado ou alucinações, perda do reconhecimento de locais familiares e perda de interesse e incapacidade de realizar as atividades habituais.

Demência vascular: qualquer demência causada por doença cerebrovascular, utilizado para descrever os efeitos de grandes lesões tromboembólicas.

Demência por Corpos de Lewy: é uma demência do tipo Alzheimer com perda progressiva da função mental, caracterizada pelo desenvolvimento de corpos de Lewy nas células nervosas causando declínio cognitivo com alucinações visuais recorrentes e flutuação no estado cognitivo.

Demência pela doença de Parkinson: é a perda da função mental caracterizada pelo desenvolvimento de corpos de Lewy em pessoas que têm a doença de Parkinson.

Demência fronto-temporal: caracteriza-se por significativas modificações do comportamento e da personalidade, enquanto o processo de aprendizado e elaboração de conhecimento encontra-se relativamente preservado entre 45 e 65 anos de idade. Sinais e sintomas: apatia (sentir-se indiferente ou sem emoção e/ou desinteressado), desinibição (que não demonstra timidez) e comportamentos inadequados (por exemplo, fazer algo além de um ponto apropriado e/ou repetição inadequada de comportamento, som palavra e/ou frase) ou estereotipados (comportamentos motores ou verbais repetidos). A linguagem, por sua vez, é progressivamente afetada, podendo ocorrer dificuldades na compreensão e na expressão verbal, com redução da fluência ou mesmo mutismo.

Demência de Huntington: é uma doença genética neurodegenerativa caracterizada por sintomas psiquiátricos, motores e cognitivos progressivos, como agitação, sintomas depressivos, delírios e alucinações.

Demência alcoólica: é uma forma de demência originada pelo consumo excessivo de bebidas alcoólicas a curto prazo ou a longo prazo, o que resulta em danos no cérebro e danos da função cognitiva, como por exemplo: dificuldade para aquisição de novos conhecimentos, problemas na memória a curto-prazo, alterações da personalidade, dificuldades no pensamento lógico, fracas competências sociais, problemas de equilíbrio, entre outros.

Demência de Creutzfeldt-Jakob: caracterizada por demência rapidamente progressiva, mudança de comportamento e anormalidades no modo de caminhar (por exemplo: perda de velocidade, suavidade, simétrica ou perda de equilíbrio) tendência à queda, dificuldade para manter-se em pé, realizar movimentos rápidos e/ou manter o foco e tremores acentuados, e apresenta espasmos involuntários em diversos músculos do corpo.

Doença de Alzheimer: é um transtorno neurodegenerativo progressivo e fatal que se manifesta pela deterioração cognitiva e da memória com comprometimento progressivo das atividades da rotina diária. Sinais e sintomas: falta de memória para acontecimentos recentes, repetição da mesma pergunta várias vezes, dificuldade para acompanhar conversas ou pensamentos complexos, dificuldade para dirigir automóvel e encontrar caminhos conhecidos, dificuldade para encontrar palavras que exprimem ideias ou sentimentos pessoais, irritabilidade, suspeição injustificada, agressividade, passividade, interpretações erradas de estímulos visuais ou auditivos.

Parkinson: é uma doença neurológica que afeta os movimentos do indivíduo. Sinais e sintomas: tremores, lentidão de movimentos, caminhar arrastando os pés, postura inclinada para frente, rigidez muscular, redução da quantidade de movimentos, distúrbios da fala, dificuldade para engolir, dores, tontura, distúrbios do sono, respiratórios, urinários, desequilíbrio, além de alterações na fala e na escrita.

Acidente Vascular Cerebral (AVC) ou derrame: acontece quando vasos que levam sangue ao cérebro entopem ou se rompem, provocando a paralisia da área cerebral que ficou sem circulação sanguínea. Sinais e sintomas: fraqueza ou formigamento na face, no braço ou na perna, especialmente em um lado do corpo, confusão mental, alteração da fala ou compreensão, alteração na visão, alteração do equilíbrio, coordenação, tontura ou alteração no andar, dor de cabeça súbita, intensa, sem causa aparente.

AVC transitório ou pequeno derrame transitório (com reversão dos sintomas em menos de 24 horas do evento): é diferente do AVC, pois apresenta sintomas e a recuperação completa dura menos de 1 hora, sem deixar lesões permanentes.

Trauma cranioencefálico: é qualquer lesão decorrente de um trauma que tenha como consequência fratura ou laceração do couro cabeludo, comprometimento funcional e cognitivo resultando em alterações cerebrais, momentâneas ou permanentes. Sinais e sintomas: desmaio, perda de memória, dor de cabeça intensa, dificuldade para enxergar ou perda da visão, confusão mental, vômitos, fala alterada, perda de equilíbrio, sangramentos graves na cabeça ou rosto, saída de sangue ou de líquido transparente pelo nariz e ouvidos, olho roxo, perda de sensibilidade em alguma parte do corpo e sonolência excessiva.

Hemorragia cerebral: é um tipo de acidente vascular cerebral (AVC), em que ocorre sangramento ao redor ou dentro do cérebro por conta do rompimento de um vaso sanguíneo, geralmente uma artéria do cérebro, por exemplo, uma pancada na cabeça. Sinais e sintomas: estado de inconsciência profunda, sensação de náusea, vômito, diminuição da frequência cardíaca e perda do equilíbrio.

Hematoma subdural: coleção de sangue no espaço subdural, decorrente do rompimento de veias e vasos. Normalmente associadas a edema cerebral, lesão axonal difusa (LAD) e contusões.

Tumor cerebral: é caracterizado pelo crescimento acelerado de células que sofreram uma mutação localizada no cérebro ou nas meninges (membranas que recobrem o cérebro e ficam no interior do crânio), os tumores podem ser benignos, mas em grande maioria são malignos. Sinais e sintomas: dores de cabeça, náuseas e vômitos, alterações visuais, convulsões, perda do equilíbrio, audição e sensibilidade, mudanças de humor, personalidade, capacidade de falar e articular as palavras.

Doença Pulmonar Obstrutiva Crônica (DPOC): é uma doença pulmonar inflamatória que obstrui as vias aéreas, tornando a respiração difícil. Sinais e sintomas: falta de ar aos esforços, pigarro, tosse crônica, tosse com secreção e que piora pela manhã.

Enfisema: é caracterizado pela destruição dos alvéolos no pulmão, dificultando a passagem do ar e a oxigenação do sangue. Sinais e sintomas: chiado, tosse, respiração ofegante, falta de ar.

Bronquite crônica: ocorre a inflamação dos brônquios, estruturas pulmonares responsáveis por levar e trazer o ar a cada respiração causando o estreitamento das vias aéreas e o acúmulo de secreção dentro delas. Sinais e sintomas: tosse com expectoração (secreção de muco), chiado, falta de ar e cansaço.

Asma (bronquite asmática): é uma doença que causa inflamação crônica nas vias aéreas, onde essas vias aéreas respondem aos estímulos internos e/ou externos, fazendo com que essas vias se contraiam. Associada aos eventos imunes locais, gera uma inflamação alérgica das vias aéreas. Sinais e sintomas: tosse, sibilos (miados de gato), falta de ar, opressão torácica.

Angina (dor no peito ou isquemia ou má circulação no coração): caracterizada por dor ou desconforto em qualquer das regiões como peito, parte superior do abdome, face, ombro, costas ou membros braços, sendo tipicamente desencadeada ou agravada com atividade física ou estresse emocional. A isquemia é uma deficiência no aporte sanguíneo a determinado órgão ou tecido causados pela diminuição do espaço que passa o sangue nas artérias, arteríolas e capilares.

Infarto do miocárdio: é a morte de células cardíacas, causada pela diminuição do espaço que passa o sangue nas artérias, arteríolas e capilares de forma prolongada. Sinais e sintomas: Dor no peito em aperto à esquerda, que se espalha para o braço esquerdo, de grande intensidade e prolongada (>20 minutos), melhorando parcialmente ou não em repouso ou com uso de medicações. A dor pode se espalhar para mandíbula, braço direito, costas, ombros e parte superior do abdome, assim como em pacientes diabéticos, idosos ou no período pós-operatório, o infarto pode ocorrer na ausência de dor, mas com náuseas, mal-estar, falta de ar, aumento da frequência cardíaca ou até confusão mental.

Cirurgia de ponte de safena: a principal finalidade desta cirurgia é revascularizar o músculo cardíaco, chamado de miocárdio, criando uma nova rota de sangue e uma vez que o volume e a pressão do sangue são restaurados pelo procedimento cirúrgico, aliviam-se sintomas das doenças cardíacas, tais como falta de ar e angina.

Cirurgia de artéria mamária: procedimento cirúrgico envolvendo a artéria mamária.

Angioplastia: é o tratamento não cirúrgico das obstruções das artérias do coração por meio de um cateter, com o objetivo de aumentar o fluxo de sangue para o coração.

Implantação de stent no coração: implante de uma prótese dentro dos vasos conhecida como 'stent' – pequeno tubo colocado dentro de uma artéria, usado para manter a artéria aberta, restaurando ou evitando a diminuição do fluxo sanguíneo por entupimento e mantendo a oxigenação dos órgãos.

Artrite: inflamação que ocorre na articulação ou articulações.

Diabetes: inclui um grupo de doenças metabólicas caracterizadas por hiperglicemia, causado por defeitos na secreção de insulina e/ou em sua ação. Sinais e sintomas: aumento da quantidade de vezes de ida ao banheiro para urinar, sede anormal ou excessiva, perda de peso, fome excessiva ou extrema e visão turva.

Insuficiência renal crônica: é definida pela lesão da estrutura renal e/ou pela diminuição funcional renal presentes por um período igual ou superior a três meses**.** Sinais e sintomas: perda de proteína pela urina, hipervolemia (volume excessivo de líquidos), anemia, dislipidemia, acidose metabólica.

Hipertensão arterial sistêmica: é uma doença crônica não transmissível definida por níveis pressóricos, caracterizada por elevação persistente da pressão arterial (PA), ou seja, PA sistólica (PAS) maior ou igual a 140 mmHg e/ou PA diastólica (PAD) maior ou igual a 90 mmHg, medida com a técnica correta, em pelo menos duas ocasiões diferentes, na ausência de medicação anti-hipertensiva. Sinais e sintomas: sinais neurológicos focais, alterações visuais, congestão, dor torácica, insuficiência renal e hepática. Diagnóstico: devem ser realizadas medidas de consultório, o diagnóstico de HA deverá ser sempre validado por medições repetidas, em condições ideais, em duas ou mais visitas médicas em intervalo de dias ou semanas; ou de maneira mais assertiva, realizando-se o diagnóstico com medidas fora do consultório (se não contraindicado) e desde que não haja suspeita de HA do avental branco (aumento de PAS ou PAD isolada).

Insuficiência cardíaca: é um conjunto de sinais e sintomas complexos, na qual o coração é incapaz de bombear sangue de forma a atender às necessidades do metabolismo. Sinais e sintomas: variam conforme estágio da doença e do tipo de insuficiência cardíaca, falta de ar em repouso e/ou aos esforços, cansaço e/ou falta de energia, tosse seca ou com presença de muco "espumoso" ou catarro, inchaço em pés e pernas, dificuldade para dormir, causado por falta de ar, inchaço abdominal, aumento de área cardíaca, aumento do tamanho do fígado, confusão mental.

Câncer: é o nome geral dado a um conjunto de mais de 100 doenças, que têm em comum o crescimento desordenado de células, que tendem a invadir tecidos e órgãos vizinhos.

Carcinoma basocelular em pele ou equivalente: responsável por 70% dos diagnósticos, sendo a neoplasia maligna mais comum em humanos. Apresenta comportamento invasivo e baixo potencial metastático, sendo facilmente tratável pela excisão cirúrgica, desde que diagnosticado precocemente. As neoplasias malignas ou tumores malignos são capazes de invadir tecidos vizinhos e provocar metástases, podendo ser resistentes ao tratamento e causar a morte.
Marcapasso cardíaco: é um sistema de estimulação artificial do coração que transmite estímulos elétricos por meio de um gerador de pulsos e um eletrodo com o propósito de monitorar continuamente e caso detecte um batimento cardíaco lento, ele envia pequenos sinais elétricos indetectáveis para corrigir.

Desfibrilador interno: um equipamento implantável totalmente automático, capaz de detectar arritmias graves e tratá-las imediatamente através de estímulos elétricos.

Clipe de aneurisma: o aneurisma é uma dilatação anormal de um vaso sanguíneo cujas paredes estão enfraquecidas, dependendo do caso, a intervenção cirúrgica é um procedimento antigo, porém necessário, onde um clipe é implantado na boca do aneurisma com a ideia de bloquear a circulação sanguínea e evitar seu rompimento.

Expansor mamário: é um balão feito de silicone, como as próteses, preenchidos gradativamente com soro fisiológico, até a obtenção do volume desejado.

Prótese mamária: são implantes colocados nos seios para um aumento do volume na região.

Válvula cerebral: é um tubo que regula a quantidade, direção de fluxo e pressão do líquido cefalorraquidiano para fora dos ventrículos cerebrais.

Próteses (ortopédica, peniana, ouvido, ocular, cardíaca): é uma peça artificial que substitui uma parte do corpo.

Traqueostomia metálica: é um procedimento cirúrgico que consiste em uma abertura realizada na traqueia, com inserção de um tubo metálico, que irá permitir a passagem do ar.

Parkinson: é uma doença neurológica que afeta os movimentos do indivíduo. Sinais e sintomas: tremores, lentidão de movimentos, caminhar arrastando os pés, postura inclinada para frente, rigidez muscular, redução da quantidade de movimentos, distúrbios da fala, dificuldade para engolir, dores, tontura, distúrbios do sono, respiratórios, urinários, desequilíbrio, além de alterações na fala e na escrita.

Claustrofobia: é uma fobia ou medo exagerado e irracional de permanecer em ambientes fechados ou com pouca circulação de ar, que pode ser desde elevadores, salas pequenas ou transporte público.

Hidrocefalia: no interior do cérebro existem espaços chamados de ventrículos, que são preenchidas pelo líquido cefalorraquidiano (LCR) ou líquor, a hidrocefalia acontece quando a quantidade desse líquido aumenta dentro do crânio comprimindo o cérebro provocando uma série de sintomas que necessitam de tratamento de emergência para prevenir danos mais sérios.

Referências

1. Campos Hisbello S. Asma: suas origens, mecanismos inflamatórios e o papel do corticosteroide. Rev Bras Pneumol Higienizar [Internet]. 2007 Dez;15(1):47-60. Disponível em: http://scielo.iec.gov.br/scielo.php?script=sci_arttext&pid=S1982-32582007000100007&lng=pt.

2. Mansur AP de P, Armaganijan D, Amino JG, Sousa AC, Simão AF, Brito AX de, et al. Diretrizes de doença coronariana crônica angina estável. Arq Bras Cardiol [Internet]. 2004 Sep;83(suppl 2):2-43. Disponível em: https://doi.org/10.1590/S0066-782X2004002100001.

3. Vasconcelos AC. Patologia Geral em Hipertexto. Universidade Federal de Minas Gerais. Belo Horizonte, Minas Gerais; 2000.

4. Pereira PAE, Serrano JCV, Carlos NJ. Infarto Agudo do Miocárdio - Síndrome Coronariana Aguda com supradesnível do segmento ST. Rev Assoc Med Bras [Internet]. 2004 Feb 26. Disponível em: https://www.scielo.br/j/ramb/a/kKY84ZFgn3Jjx8Dv9dMsh8p/?format=pdf&lang=pt.

5. Ferreira JP, Ferreira CSB, Ruschel PP, Petrarca RCT. Qualidade de vida em pacientes pós-operatórios de cirurgia cardíaca. Rev SBPH [Internet]. 2013 Dez [citado 2023 Mar 08];16(2):120-136. Disponível em: http://pepsic.bvsalud.org/scielo.php?script=sci_arttext&pid=S1516-08582013000200009&lng=pt.

6. Mesquita NRMC, Basílio BR, Moreira CFS. Revascularização cirúrgica do miocárdio com uso de enxerto autólogo de artéria torácica interna. Rev Méd Minas Gerais [Internet]. 2016. Disponível em: https://rmmg.org/artigo/detalhes/1971.

7. Feres F, Costa RA, Siqueira D, Costa JR, Chamié D, Staico R, et al. Diretriz da Sociedade Brasileira de Cardiologia e da Sociedade Brasileira de Hemodinâmica e Cardiologia Intervencionista sobre intervenção coronária percutânea. Arq Bras Cardiol [Internet]. 2017 Jul;109(suppl 1):1-81. Disponível em: https://doi.org/10.5935/abc.20170111.

8. Gross JL, Silveiro SP, Camargo JL, Reichelt AJ, Azevedo MJ de. Diabetes Melito: Diagnóstico, Classificação e Avaliação do Controle Glicêmico. Arq Bras Endocrinol Metab [Internet]. 2002 Feb;46(1):16-26. Disponível em: https://doi.org/10.1590/S0004-27302002000100004.

9. Bastos MG, Bregman R, Kirsztajn GM. Doença renal crônica: frequente e grave, mas também prevenível e tratável. Rev Assoc Med Bras [Internet]. 2010;56(2):248-53. Disponível em: https://doi.org/10.1590/S0104-42302010000200028.

10. Barroso WKS, Rodrigues CIS, Bortolotto LA, Mota-Gomes MA, Brandão AA, Feitosa ADM, et al. Diretrizes Brasileiras de Hipertensão Arterial – 2020. Arq Bras Cardiol [Internet]. 2021;116(3):516-658. Disponível em: http://departamentos.cardiol.br/sbc-dha/profissional/pdf/Diretriz-HAS-2020.pdf.

11. Diretriz Brasileira de Insuficiência Cardíaca Crônica e Aguda. Arq Bras Cardiol [Internet]. 2018 Sep;111(3):436-539. Disponível em: https://doi.org/10.5935/abc.20180190.

12. ABC do câncer: abordagens básicas para o controle do câncer [Internet]; 2011. UNIDADE I: O QUE É O CÂNCER, ABC do câncer: abordagens básicas para o controle do câncer; p. 17. Disponível em: https://bvsms.saude.gov.br/%20bvs/controle_cancer.

13. Brasil. Ministério da Saúde. Biblioteca Virtual em Saúde. 30/8 – Dia Nacional de Conscientização Sobre a Esclerose Múltipla. Brasília: Ministério da Saúde. Disponível em: https://bvsms.saude.gov.br/30-8-dia-nacional-de-conscientizacao-sobre-a-esclerose-multipla/.

14. Brasil. Ministério da Saúde. Saúde de A a Z. Esclerose Lateral Amiotrófica (ELA). Brasília: Ministério da Saúde. Disponível em: https://www.gov.br/saude/pt-br/assuntos/saude-de-a-a-z/e/ela.

15. Brasil. Ministério da Saúde. Dia mundial de conscientização. Epilepsia: conheça a doença e os tratamentos disponíveis no SUS. Brasília: Ministério da Saúde. 2022. Disponível em: https://www.gov.br/saude/pt-br/assuntos/noticias/2022/marco/epilepsia-conheca-a-doenca-e-os-tratamentos-disponiveis-no-sus.

16. BVS Atenção Primária em Saúde. O que é demência? Núcleo de Telessaúde Rio Grande do Sul. Dez 2009. Disponível em: https://aps-repo.bvs.br/aps/o-que-e-demencia/#:~:text=Os%20principais%20sinais%20e%20sintomas,de%20realizar%20as%20atividades%20habituais.

17. Biblioteca Virtual em Saúde. Ministério da Saúde. Doença de Parkinson. Brasília: Ministério da Saúde. 2012. Disponível em: https://bvsms.saude.gov.br/doenca-de-parkinson/.

18. Brasil. Ministério da Saúde. Saúde de A a Z. Acidente Vascular Cerebral. Brasília: Ministério da Saúde. Disponível em: https://www.gov.br/saude/pt-br/assuntos/saude-de-a-a-z/a/avc.

19. Brasil. Ministério da Saúde. Saúde do cérebro. AIT: Ataque Isquêmico Transitório pode evoluir para AVC, alertam especialistas. Brasília: Ministério da Saúde. 2023. Disponível em: https://www.gov.br/saude/pt-br/assuntos/noticias/2023/janeiro/ait-ataque-isquemico-transitorio-pode-evoluir-para-avc-alertam-especialistas#:~:text=Diferente%20do%20Acidente%20Vascular%20Cerebral,ser%20avaliado%20por%20um%20m%C3%A9dico.

20. Brasil. Ministério da Saúde. Secretaria de Atenção à Saúde. Departamento de Ações Programáticas Estratégicas. Diretrizes de Atenção à Reabilitação da Pessoa com Traumatismo Cranioencefálico. Brasília: Ministério da Saúde. 2015.

21. Teixeira-Jr AL, Salgado JV. Demência fronto-temporal: aspectos clínicos e terapêuticos. Rev Psiquiatr Rio Gd Sul [Internet]. 2006 Jan;28(1):69-76. Disponível em: https://doi.org/10.1590/S0101-81082006000100009.

22. Coelho VAA, Beato RG, Prado PHT do, Cardoso FEC, Lauar H. Doença de Huntington: relato de caso com história familiar negativa e atenuação das manifestações psiquiátricas com o uso de olanzapina. Braz J Psychiatry [Internet]. 2009 Jun;31(2):186-8. Disponível em: https://doi.org/10.1590/S1516-44462009000200020.

23. Demências [Internet]. [place unknown]; 2021. Demências; [cited 2023 Apr 3]. Disponível em: https://clinicajorgejaber.com.br/novo/wp-content/uploads/2021/11/Deme%CC%82ncias.pdf.

24. Reis F, Palma ALG, Schwingel R, Torres HHJ, Oshima MM, Queiroz LS, et al. Creutzfeldt-Jakob dementia. Radiol Bras [Internet]. 2015 Jul;48(4):267-8. Disponível em: https://doi.org/10.1590/0100-3984.2014.0109.

25. ANDRÉ C. Demência vascular: dificuldades diagnósticas e tratamento. Arq Neuro-Psiquiatr [Internet]. 1998 Sep;56(3A):498-510. Disponível em: https://doi.org/10.1590/S0004-282X1998000300025.

26. Ministério da Saúde (BR). A doença de Alzheimer. [Internet]. Brasília, DF: Ministério da Saúde (BR); c2021. Disponível em: https://www.gov.br/saude/pt-br/assuntos/saude-de-a-a-z/a/alzheimer.

27. Manual MSD [Internet]. Distrúrbios cerebrais, da medula espinal e dos nervos: delirium e demência: Demência por corpos de Lewy e demência da doença de Parkinson. Disponível em: https://www.msdmanuals.com/pt-br/casa/distúrbios-cerebrais,-da-medula-espinal-e-dos-nervos/delirium-e-demência/demência-por-corpos-de-lewy-e-demência-da-doença-de-parkinson.

Anexo 4. POP Enfermagem em urgências e emergências

	**PROCEDIMENTO OPERACIONAL PADRÃO**
**Área:** ELSA-Brasil	**Página**: 1/9
**Assunto:** Enfermagem em urgências e emergências - ELSA Cérebro	**Vigência:** 10/05/2023

1. OBJETIVO

Estabelecer os critérios para orientar as ações referentes ao atendimento às principais urgências e emergências no ambiente de ressonância magnética, baseados nos sinais e sintomas, pressão arterial sistêmica, frequência e oximetria de pulso.

2. RESPONSABILIDADES

2.1 Enfermeiro de pesquisa

3. DEFINIÇÕES

3.1 **Urgência:** É todo o quadro de saúde que implique sofrimento intenso, exigindo, portanto, tratamento imediato com o mínimo tempo de espera possível, mesmo sem risco iminente de morte. Assim, o atendimento rápido serve para que os pacientes não sofram complicações e tenham uma melhora dos sintomas rapidamente.

3.2 **Emergência:** Caracterizado como o risco iminente de morte e a necessidade de adotar rapidamente medidas diagnósticas e terapêuticas de suporte à vida no qual é necessário ser atendidas por um time de profissionais de saúde de forma rápida e eficaz, preferencialmente em um ambiente com recursos médicos para suporte de vida.

4. DESCRIÇÃO

4.1 **Crise hipertensiva:** A hipertensão arterial sistêmica (HAS) é uma condição clínica multifatorial caracterizada por níveis elevados e sustentados de pressão arterial (PA). Associa-se frequentemente a alterações funcionais e/ou estruturais dos órgãos-alvo (coração, encéfalo, rins e vasos sanguíneos) e a alterações metabólicas, com consequente aumento do risco de eventos cardiovasculares fatais e não fatais.

4.2 **Características clínicas**

Emergência Hipertensiva (EH) é definida pela elevação dos níveis pressóricos associada à lesão aguda em órgãos-alvo ou risco iminente de morte; requer internação em unidade de cuidados intensivos e redução imediata da PA, a redução inicial não deve ultrapassar 20% a 25% da PA média inicial.

Urgência Hipertensiva (UH) é definida pela elevação da pressão arterial sem lesão de órgão-alvo, geralmente associada à interrupção ou redução da medicação anti-hipertensiva. Pacientes com urgência hipertensiva devem ter sua pressão arterial reduzida em 24 a 48 horas. Manifestações clínicas: angina instável, anticoagulação, intoxicação por cocaína ou anfetamina, pré ou pós-operatório, transplante renal, rebote hipertensivo após suspensão súbita de medicações anti-hipertensivas.

4.3 **Classificação da pressão arterial**

Quadro 1 - Classificação da pressão arterial de acordo com a medição no consultório a partir de 18 anos de idade[1]

**Classificação**	**PAS (mmHg)**		**PAD (mmHg)**
PA ótima	<120	e	<80
PA normal	120 – 129	e/ou	80 – 84
Pré-hipertensão	130 – 139	e/ou	85 – 89
HA estágio 1	140 – 159	e/ou	85 – 89
HA estágio 2	160 – 179	e/ou	100 – 109
HA estágio 3	>= 180	e/ou	>= 110

Referência

1. Barroso WKS, Rodrigues CIS, Bortolotto LA, Mota-Gomes MA, Brandão AA, Feitosa ADM, Machado CA, et al. Diretrizes Brasileiras de Hipertensão Arterial – 2020. Arq Bras Cardiol. 2021;116(3):516-658. Disponível em: http://departamentos.cardiol.br/sbc-dha/profissional/pdf/Diretriz-HAS-2020.pdf.

4.4 **Ações Técnicas de Enfermagem**

Anamnese: História prévia de HAS, tratamento atual, sintomas de disfunções cardíacas, cerebrais, visuais e/ ou renais (cefaleia, dispneia, angina, alteração do nível de consciência).

Conduta: Se hipertensão arterial sistêmica sintomática, comunicar ao biomédico e a enfermagem do setor.

5. DESCRIÇÃO

5.1 Hipotensão arterial: ocorre quando a pressão arterial cai a ponto de provocar sintomas como tonturas e desmaios, sendo que pode causar danos a órgãos, um processo chamado choque.

5.2 Hipotensão postural: é mais comum nos pacientes idosos com hipertensão sistólica, caracteriza-se por decréscimo de mais de 10 mm Hg na pressão sistólica na posição ortostática, e associa-se a tontura ou síncope.

5.3 Ações técnicas de Enfermagem

Anamnese: História prévia de hipotensão, quedas, tratamento atual, uso de diuréticos, vasodilatadores (nitratos, sildenafila e fármacos com mecanismo de ação semelhante) e alguns psicotrópicos

Conduta: checar sinais vitais e comunicar ao biomédico e a enfermagem do setor.

6. DESCRIÇÃO

6.1 Hipoxemia: é caracterizada pela baixa quantidade de oxigênio transportado pelo sangue, levando à hipóxia, condição na qual a quantidade de oxigênio ofertada não é suficiente para uma adequada respiração celular e as consequências vão desde sintomas e alterações fisiológicas relacionadas à tentativa do corpo a se adaptar à baixa concentração de oxigênio, falhas em funções essenciais do corpo, até a morte de células, tecidos e órgãos, o que pode levar à morte do indivíduo. Há diversos fatores que podem levar ao quadro de hipoxemia.

6.2 Sinais e sintomas

- Esforço respiratório e/ou redução da concentração de oxigênio no sangue
- Batimento de asa de nariz
- Uso de musculatura acessória à respiração
- Presença de tiragens intercostais e subcostais
- Cianose central e/ou periférica
- Sensação de dispneia
- Alteração da frequência respiratória e/ou alteração do ritmo respiratório

6.3 Ações técnicas de Enfermagem

Conduta: se saturação periférica de oxigênio não basal <94% aferida por oximetria de pulso, associada ou não à presença de sintoma(s) de esforço respiratório, comunicar ao biomédico e a enfermagem do setor para devidas providências.

7. DESCRIÇÃO

7.1 Bradisfigmia: é caracterizada pela presença de pulso lento, fino e bradicárdico, diminuição da frequência de pulso, abaixo dos valores de normalidade (em geral, <60 pulsações por minuto). Possíveis sinais e sintomas: precordialgia, dispneia, queda do nível de consciência, fraqueza, fadiga, tontura, síncope, pré-sincope; sinais possíveis: hipotensão (postural ou não), diaforese, congestão pulmonar e sistêmica. Pode ser assintomática/ sinusal.

7.2 Taquicardia sinusal (FC > 100 bpm): Pode ser secundária a febre, exercícios físicos, emoções fortes (susto, medo, ansiedade, sensação de felicidade ou medo intenso), hipoxemia, dor, anemia, choque, hipertireoidismo e resposta a medicamentos.

7.3 Ações técnicas de Enfermagem

Conduta: se bradisfigmia, identificar se existem critérios de instabilidade hemodinâmica - diminuição do nível de consciência; congestão pulmonar; choque ou precordialgia, acionar imediatamente o biomédico e a enfermagem do setor para chamar médico e/ou código amarelo. Nesse caso ele (ela) não poderá realizar o exame de ressonância magnética.

Conduta: se taquicardia sinusal sintomática (palpitação cardíaca, pulso acelerado, sensação de nó na garganta, tontura, desmaio/síncope, sensação de fraqueza, cansaço, falta de ar, dor no peito e mal-estar) acionar imediatamente o biomédico e a enfermagem do setor para chamar médico e/ou código amarelo. Nesse caso ele (ela) não poderá realizar o exame de ressonância magnética.

Código amarelo HC/ InRad

Acionar quando houver pelo menos 1 critério presente:

- FR <5ipm ou FR >36ipm
- FC <50bpm ou FC>130bpm
- PAS <90mmHg
- Diminuição do nível de consciência
- Suspeita de AVC (desvio de rima labial, disartria e/ou hemiparesia)
- Preocupação com sinais e sintomas não habituais do paciente

Conduta: comunicar biomédico, enfermagem e médico do setor para avaliação. O médico acionará o TRR se julgar necessário.

Como acionar o código amarelo? Pressionando o botão amarelo ou ligando no ramal 3375/3376

Código azul HC/ InRad
Acionar em situação de Parada Cardiorrespiratória (PCR)
Como acionar? Botão azul ou ramais 3375/3376
Conduta: manobras de ressuscitação cardiopulmonar

C – Cheque responsividade e respiração
Chame ajuda e peça um DEA
Cheque pulso carotídeo
Compressões (30)
A – Abra a via aérea
B – Ventilação (2)

Referências

1. Brasil. Portaria 354, 10 de março de 2014. Proposta de Projeto de Resolução "Boas Práticas para Organização e Funcionamento de Serviços de Urgência e Emergência, portaria número 354. São Paulo, SP, Anexo 2.1 e 2.2. 01 de janeiro, 2023.

2. Neto JB, Pavão MLRC, Miranda CH. Bradiarritimias. Rev QualidadeHC. 2017.

3. Ministério da Saúde. Linha de cuidado do adulto com hipertensão arterial sistêmica. Ministério da Saúde, Secretaria de Atenção Primária à Saúde, Departamento de Saúde da Família [Internet]. 2021 [citado 2023 Abr 4]. Disponível em: http://bvsms.saude.gov.br/bvs/publicacoes/linha_cuidado_adulto_hipertensão_arterial.pdf

4. Kohlmann Jr O, Costa Guimarães A, Carvalho MHC, Chaves Jr HC, Machado CA, Praxedes JN, et al. III Consenso Brasileiro de Hipertensão Arterial. Arq Bras Endocrinol Metab [Internet]. 1999 Ago;43(4):257-86. Disponível em: https://doi.org/10.1590/S0004-27301999000400004

5. Quintanilha HL, Miranda. Suplementação de oxigênio no atendimento pré-hospitalar (APH). Corpo de Bombeiros Militar do Distrito Federal Departamento de Ensino, Pesquisa, Ciência e Tecnologia Direção de Ensino. 2022 Nov 17.

6. Protocolos emergência e urgência. [place unknown: publisher unknown]; 2017. Protocolos emergência e urgência.

**Elaborado por equipe do Estudo de Envelhecimento e Funcionamento Cerebral do ELSA- Brasil**	10/05/23

Anexo 5. POP Técnica para aferição de pressão arterial (PA)

**PROCEDIMENTO OPERACIONAL PADRÃO**	
**Área:** ELSA-Brasil	**Página**: 1/8
**Assunto:** Técnica para aferição de pressão arterial (PA)	**Vigência:** 10/05/2023

1. OBJETIVO

Estabelecer os critérios e as práticas para orientar as ações referente à aferição da pressão arterial por método auscultatório e oscilométrico.

2. RESPONSABILIDADES

2.1. Enfermeiro de pesquisa

3. DEFINIÇÕES

3.1. A hipertensão arterial (HA) é uma doença crônica não transmissível (DCNT) definida por níveis pressóricos, em que os benefícios do tratamento (não medicamentoso e/ ou medicamentoso) superam os riscos. Trata-se de uma condição multifatorial, que depende de fatores genéticos/epigenéticos, ambientais e sociais, caracterizada por elevação persistente da pressão arterial (PA), ou seja, PA sistólica (PAS) maior ou igual a 140 mmHg e/ou PA diastólica (PAD) maior ou igual a 90 mmHg, medida com a técnica correta, em pelo menos duas ocasiões diferentes, na ausência de medicação anti-hipertensiva. É aconselhável, quando possível, a validação de tais medidas por meio de avaliação da PA fora do consultório por meio da Monitorização Ambulatorial da Pressão Arterial (MAPA), da Monitorização Residencial da Pressão Arterial (MRPA) ou da Automedida da Pressão Arterial (AMPA).

4. DESCRIÇÃO DOS PROCEDIMENTOS

4.1. **Aferição da pressão arterial (PA)**

4.1.1. **Material**

- Ficha ou documento para registro, papel e caneta;
- Fita métrica inelástica e flexível;
- Esfigmomanômetro com manguito adequado;
- Estetoscópio;
- Dispositivo eletrônico certificado pelo INMETRO para medição de PA;
- Cadeira com encosto para as costas;
- Local para acomodação do membro superior;
- Álcool 70%;
- Bolas de algodão.

4.2. **Ações Técnicas de Enfermagem**

Figura 1 - Posicionamento do manguito na artéria braquial

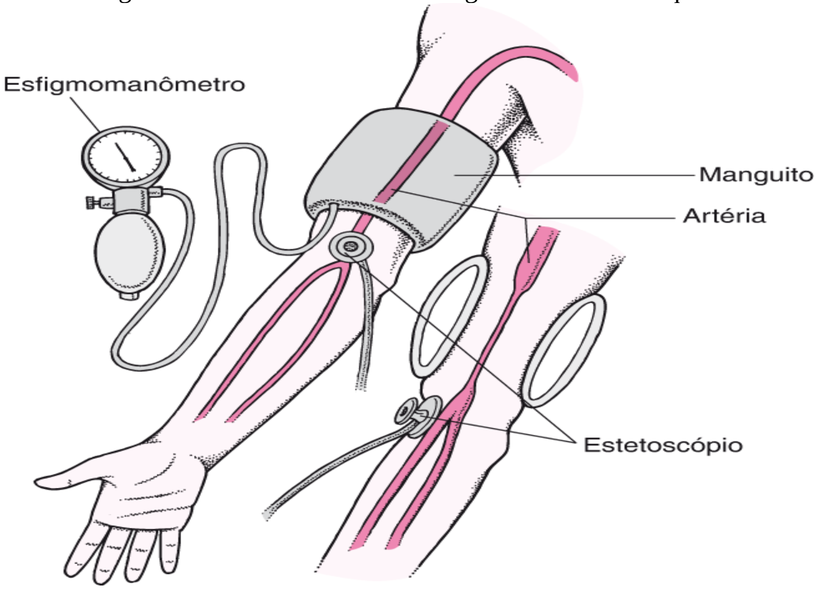

Fonte: (Bakris, s.d.). Imagem retirada de website Manual MSD. Disponível em: https://www.msdmanuals.com/pt-pt/casa/multimedia/figure/medi%C3%A7%C3%A3o-da-press%C3%A3o-arterial

4.2.1. **Técnica auscultatória**

1. Realizar a higiene das mãos;
2. Apresentar-se e explicar o procedimento a ser realizado ao participante;
3. Manter o participante em posição sentado, com o antebraço apoiado na mesa, na altura do precórdio, com a palma da mão voltada para cima, cotovelo ligeiramente fletido e pernas descruzadas.
3. Determinar a circunferência do braço com fita métrica no ponto médio entre o acrômio e o olécrano;
4. Selecionar o manguito de tamanho adequado ao braço;
5. Colocar o manguito, sem deixar folgas, 2 a 3 cm acima da fossa cubital;
6. Centralizar o meio da parte compressiva do manguito sobre a artéria braquial;
7. Estimar o nível da PAS pela palpação do pulso radial;
8. Palpar a artéria braquial em fossa cubital e colocar a campânula ou o diafragma do estetoscópio sem compressão excessiva;
9. Inflar rapidamente até ultrapassar 20 a 30 mmHg o nível estimado da PAS obtido pela palpação;
10. Proceder à deflação lentamente (velocidade de 2 mmHg por segundo);
11. Determinar a PAS pela ausculta do primeiro som (fase I de Korotkoff) e, depois, aumentar ligeiramente a velocidade de deflação;
12. Determinar a PAD no desaparecimento dos sons (fase V de Korotkoff);
13. Auscultar cerca de 20 a 30 mmHg abaixo do último som para confirmar seu desaparecimento e, depois proceder, à deflação rápida e completa;
14. Se os batimentos persistirem até o nível zero, determinar a PAD no abafamento dos sons (fase IV de Korotkoff);
15. Anotar valores da PAS/PAD/zero sem arredondamentos, o braço em que a PA foi medida, a posição do paciente, o tamanho do manguito e qualquer possível interferente;
16. Informar o valor de PA para o participante.
17. Realizar assepsia nas olivas e campânula do estetoscópio e manguito com algodão e álcool 70%.

*PAS: pressão arterial sistólica; *PAD: pressão arterial diastólica.
*Verificar presença do pulso radial por palpação antes de estimar a PAS.

4.2.2. Técnica oscilométrica (aparelho automático)

1. Realizar a higiene das mãos;
2. Apresentar-se e explicar o procedimento a ser realizado ao participante;
3. Manter o participante em posição sentado, com o antebraço apoiado na mesa, na altura do precórdio, com a palma da mão voltada para cima, cotovelo ligeiramente fletido e pernas descruzadas.
4. Determinar a circunferência do braço no ponto médio entre acrômio e olécrano;
5. Selecionar o manguito de tamanho adequado ao braço;
6. Colocar o manguito, sem deixar folgas, 2 a 3 cm acima da fossa cubital;
7. Centralizar o meio da parte compressiva do manguito sobre a artéria braquial;
8. Realizar pelo menos duas medições, com intervalo em torno de um minuto.
9. Medições adicionais deverão ser realizadas se as duas primeiras tiverem 5mmHg ou mais de diferença.
10. Aguardar 1-2 minutos entre as medidas no mesmo braço;
11. Considerar a média das duas últimas medidas;
12. Informar o valor de PA obtido para o participante;
13. Anotar os valores exatos sem “arredondamentos”, o braço em que a PA foi medida, a posição do paciente, o tamanho do manguito e qualquer possível interferente.
14. Informar o valor de PA para o participante.
15. Realizar assepsia do manguito com algodão e álcool 70%.

4.2.3 Técnica para aferição de PA em membros inferiores (artéria poplítea)

1. Realizar a higiene das mãos;
2. Apresentar-se e explicar o procedimento a ser realizado ao participante;
3. Posicionar o paciente em decúbito ventral;
4. Envolver a coxa do paciente com o manguito, posicionando o meio da parte compressiva sobre a artéria poplítea, deixando o manômetro em posição visível;
5. Localizar, com os dedos indicador e médio, a artéria poplítea (palpável atrás do joelho);

6. Seguir item 7 da técnica auscultatória, estimando nível de PAS pela palpação do pulso poplíteo, alterando o pulso a ser palpado e o posicionamento da campânula para a artéria poplítea.

4.2.4 Técnica para aferição de PA em membros inferiores (artéria tibial posterior)

1. Realizar a higiene das mãos;
2. Apresentar-se e explicar o procedimento a ser realizado ao participante;
3. Posicionar o paciente em decúbito dorsal;
4. Envolver o terço inferior da perna do paciente com o manguito, posicionando o manguito 5cm acima do maléolo (proeminência óssea) do terço inferior da perna, com o meio da parte compressiva sobre a artéria tibial posterior; deixar o manômetro em posição visível;
5. Localizar, com os dedos indicador e médio, a artéria tibial posterior (face posterior do maléolo medial);
6. Seguir item 7 da técnica auscultatória, estimando nível de PAS pela palpação do pulso tibial posterior, bem como o posicionamento da campânula.

Figura 2 - Posicionamento do manguito nas artérias tibial posterior e poplítea

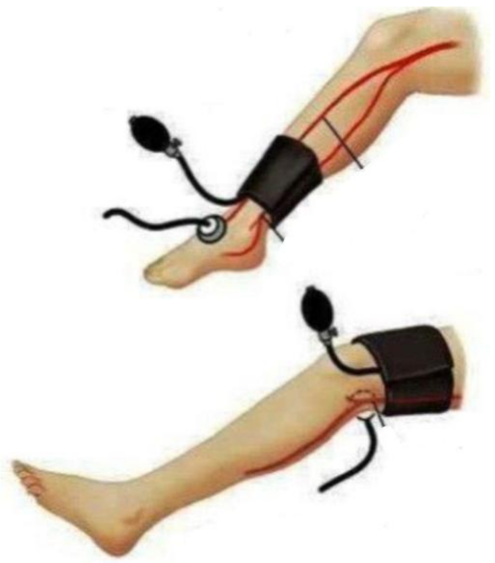

Fonte: (Brainly). Imagem retirada de website Brainly. Disponível em: https://brainly.com.br/tarefa/22437628

Pontos de Atenção

- O comprimento da bolsa inflável deve ser de 80% a 100% da circunferência do braço (CB) e a largura de, pelo menos, 40% da CB.
- O participante deve sentar-se confortavelmente em um ambiente silencioso por 5 minutos, antes de iniciar as medições da PA.
- Certificar-se de que o participante NÃO: a) Está com a bexiga cheia; b) Praticou exercícios físicos há, pelo menos, 60 minutos; c) Ingeriu bebidas alcoólicas, café ou alimentos; d) Fumou nos 30 minutos anteriores; e) Fale durante a aferição.
- Medidas adicionais podem ter que ser realizadas em participantes com valores instáveis da PA devido a arritmias. Nos pacientes com FA, os métodos auscultatórios devem ser preferencialmente usados, pois a maioria dos dispositivos automáticos não foi validada para a medida da PA.
- Use o manguito adequado para a circunferência do braço. O manguito deve ser posicionado ao nível do coração. A palma da mão deve estar voltada para cima e as roupas não devem garrotear o braço. As costas e o antebraço devem estar apoiados; as pernas, descruzadas; e os pés, apoiados no chão.
- A maioria dos dispositivos automáticos registra a forma de onda de pressão sistólica individual mais alta em vez de uma média de vários ciclos cardíacos em pacientes com FA, o que leva à superestimação da PA.
- Se o/a participante com antecedente de mastectomia bilateral, realizar aferição de PA em membros inferiores, se não contraindicado.
- Não aferir a PA em membro com presença de fístula arteriovenosa, membro hemiparético e/ou presença de cateter venoso de longa permanência.
- Observação: A pressão sistólica nas pernas é geralmente mais elevada em 10 a 40 mmHg que na artéria braquial devido ao fenômeno da amplificação do pulso distal que ocorre progressivamente

das artérias centrais para a periferia. Já a pressão diastólica é a mesma. (POTTER et al. 2018; VII Diretrizes Brasileira de Hipertensão, 2016)

- A aferição deve ser realizada com instrumento devidamente validado e calibrado.

4.3 Prevenção de Agravos (Ações para Diminuir os Riscos)

- Se o participante com antecedente de **mastectomia bilateral**, realizar aferição de PA em membros inferiores.
- Não aferir a PA em membro com presença de **fístula arteriovenosa**, **membro hemiparético**, participante com cirurgia prévia de mastectomia, e/ou presença de **cateter venoso de longa permanência**.

5. ANEXOS

5.1. Classificação da pressão arterial

Quadro 1 - Classificação da pressão arterial de acordo com a medição no consultório a partir de 18 anos de idade

Classificação	PAS (mmHg)		PAD (mmHg)
**PA ótima**	<120	e	<80
**PA normal**	120 – 129	e/ou	80 – 84
**Pré-hipertensão**	130 – 139	e/ou	85 – 89
**HA estágio 1**	140 – 159	e/ou	85 – 89
**HA estágio 2**	160 – 179	e/ou	100 – 109
**HA estágio 3**	>= 180	e/ou	>= 110

5.2. Dimensões do manguito por circunferência braquial

Quadro 2 – Dimensões do manguito por circunferência braquial

Denominação	Circunferência do braço (cm)	Manguito (cm)	
		Largura (cm)	Comprimento (cm)
**Criança**	11 – 15	6	12
**Infantil**	16 – 22	9	18
**Adulto pequeno**	20 – 26	10	17
**Adulto**	27 – 34	12	23
**Adulto grande**	35 – 45	16	32
**Coxa**	45 - 52	20	42

Referências

1. Barroso WKS, Rodrigues CIS, Bortolotto LA, Mota-Gomes MA, Brandão AA, Feitosa ADM, et al. Diretrizes Brasileiras de Hipertensão Arterial – 2020. Arq Bras Cardiol. 2021;116(3):516-658. Disponível em: http://departamentos.cardiol.br/sbc-dha/profissional/pdf/Diretriz-HAS-2020.pdf

2. Conselho Federal de Farmácia. Procedimento de medida da Pressão Arterial. Semana de conscientização sobre o uso e descarte responsável de medicamentos. 2017. Foz do Iguaçu - Paraná. Disponível em: https://www.cff.org.br/userfiles/Procedimento%20de%20medida%20da%20Press%C3%A3o%20Arterial.pdf

3. Universidade Federal do Rio de Janeiro (UFRJ). Procedimento Operacional Padrão POP N° 22. Título: Verificação da Pressão Arterial em Área de Aplicação: Obstetrícia Adultos Setor: Todos os setores. Disponível em: http://www.me.ufrj.br/images/pdfs/protocolos/enfermagem/pop_22_verificacao_pressao_arterial_adultos.pdf

4. Ministério da Saúde. Linha de cuidado do adulto com hipertensão arterial sistêmica. Ministério da Saúde, Secretaria de Atenção Primária à Saúde, Departamento de Saúde da Família [Internet]. 2021 [citado 2023 Abr 4]. Disponível em: http://bvsms.saude.gov.br/bvs/publicacoes/linha_cuidado_adulto_hipertensão_arterial.pdf

5. Kreuning EB, Graube SL, Meneghete MC, Fontana RT, Rodrigues FCP, Bittencourt VL. Protocolo de aferição da pressão arterial em membros inferiores. Rev Baiana Enferm [Internet]. 2018 [citado 2023 Abr 4];32:e27394. Disponível em: http://www.revenf.bvs.br/scielo.php?script=sci_arttext&pid=S2178-86502018000100601&lng=pt. Epub 14-fev-2019. https://dx.doi.org/10.18471/rbe.v32.27394

6. Kohlmann Jr. O, Costa Guimarães A, Carvalho MHC, Chaves Jr HC, Machado CA, Praxedes JN, et al. III Consenso Brasileiro de Hipertensão Arterial. Arq Bras Endocrinol Metab [Internet]. 1999 Ago;43(4):257-86. Disponível em: https://doi.org/10.1590/S0004-27301999000400004

**Elaborado por equipe do Estudo de Envelhecimento e Funcionamento Cerebral, ELSA- Brasil**	10/05/23

Anexo 6. POP Técnica para aferição de frequência de pulso e oximetria

	**PROCEDIMENTO OPERACIONAL PADRÃO**
**Área:** ELSA-Brasil	**Página**: 1/8
**Assunto:** Técnica para aferição de frequência de pulso e oximetria	**Vigência:** 10/05/2023

1. OBJETIVO

Estabelecer os critérios e as práticas para orientar as ações referentes à aferição da frequência de pulso e oximetria de pulso

2. RESPONSABILIDADES

2.1. Enfermeiro de pesquisa

3. DEFINIÇÕES

3.1. A frequência de pulso: o pulso arterial é percebido como uma expansão arterial síncrona com os batimentos cardíacos, na ausência de arritmia, à palpação arterial ou por sensor de pulso.

3.2. A oximetria de pulso arterial (OPA) é um método não invasivo que fornece informações de relevância clínica sobre a saturação de oxigênio carreado pelas hemoglobinas presentes no sangue arterial e permite analisar a amplitude e a frequência de pulso, tanto na fase de repouso como de atividade, de indivíduos de qualquer faixa etária. Permite medir a saturação de oxigênio (SpO_2) em uma região anatômica, preferencialmente de localização periférica, como as extremidades distais das mãos e dos pés, mãos, pés, lóbulo da orelha, dentre outros.

4. DESCRIÇÃO DOS PROCEDIMENTOS

4.1. Aferição da frequência de pulso

4.1.1. Material

- Relógio com marcador de segundos;
- Sensor de pulso
- Caneta;
- Papel para anotações

4.2. **Ações Técnicas de Enfermagem**

4.2.1. Frequência de pulso

1. Realizar a higiene das mãos;
2. Apresentar-se e explicar o procedimento a ser realizado ao participante;
3. Manter o(a) participante em posição sentado, confortável e seguro(a) com o braço apoiado;
4. Expor o local da artéria selecionada;
5. Aproximar o relógio do campo visual;
6. Colocar as polpas dos dedos indicador e médio sobre a artéria, comprimindo-a, moderadamente, até sentir a sua pulsação e contar a frequência das pulsações por um minuto;

7. Observar: estado da parede da artéria (lisa, endurecida, tortuosa), ritmo (regular, irregular), amplitude ou magnitude (ampla, mediana, pequena) e tensão;
8. Comparar com o lado homólogo (igualdade, desigualdade).
9. Anotar
10. Lavar as mãos ao término

4.2.2. Oximetria de pulso e frequência de pulso

1. Higienizar as mãos;
2. Ligar o oxímetro: ele fará calibração interna e verificações;
3. Aguardar o tempo de calibração para aferição;
4. Certificar-se de que a área onde será feita a aferição está limpa;
5. Remover o esmalte das unhas;
6. Posicionar o sensor com cuidado, preferencialmente no dedo indicador;
7. Certificar-se de que ele se adapta facilmente sem estar muito solto ou muito apertado.
8. Aguardar para que o oxímetro detecte o pulso e calcule a saturação de oxigênio;
9. Uma vez que o pulso tenha sido detectado, a saturação de oxigênio e frequência de pulso serão exibidas;
10. Ler o dado correspondente na tela do visor ou monitor e registrar;
11. Higienizar as mãos;
12. Realizar a desinfecção do oxímetro

Fatores que interferem a determinação da oximetria de pulso

- Fontes externas de luz interferem na habilidade do oxímetro de processar a luz refletida.
- Monóxido de carbono (causado pela inalação de fumaça ou envenenamento) eleva artificialmente a SpO2 por absorver luz.
- Esmaltes e unhas artificiais interferem na absorção da luz e na habilidade do oxímetro em processar a luz refletida.
- A administração de vasoconstritores diminui o volume do pulso periférico, desta forma o oxímetro pode não detectar o sinal.
- Hipotermia, hipotensão e débito cardíaco reduzido diminuem a circulação sanguínea para as artérias.
- Tremores: o movimento pode dificultar a captação do sinal pelo sensor.
- Tabagismo.
- Edema.
- Pilha fraca do oxímetro.

5. ANEXOS

5.1. Classificação da frequência de pulso

**Quadro 1 - Classificação da frequência de pulso**	
**Idade**	**Batimentos/min**
**Lactente**	120 a 160
**Criança pequena**	90 a 140
**Pré-escolar**	80 a 110
**Criança em idade escolar**	75 a 100
**Adolescente**	60 a 90
**Adulto**	60 a 100

Referências

1. Conselho Regional de Enfermagem de São Paulo. Uso da oximetria de pulso na avaliação da saturação periférica de oxigênio em pacientes com COVID-19 [Internet]. São Paulo: Conselho Regional de Enfermagem de São Paulo; 2020. Disponível em: https://portal.coren-sp.gov.br/sites/default/files/oximetria%2022-12.pdf

2. Secretaria de Estado da Saúde do Paraná. Monitorização da oximetria de pulso na atenção primária à saúde: orientações para profissionais da saúde [Internet]. Curitiba: Secretaria de Estado da Saúde do Paraná; 2020. Disponível em: https://www.saude.pr.gov.br/sites/default/arquivos_restritos/files/documento/2020-08/NO_45_MONITORIZACAO_DA_OXIMETRIA_DE_PULSO_NA_APS_V1.pdf

3. Potter P, Stockert H. Fundamentos de Enfermagem. 9th ed. Grupo GEN; 2018.

4. Pulso e frequência cardíaca [Internet]. [place unknown]; 2017 [citado 2023 Mar 29]. Disponível em: https://semioclin.files.wordpress.com/2017/03/pulso-e-frequc3aancia-cardc3adaca.pdf

**Elaborado por equipe do Estudo de Envelhecimento e Funcionamento Cerebral, ELSA- Brasil**	10/05/23

Anexo 7. POP Técnica para mensuração de perímetro cefálico

	**PROCEDIMENTO OPERACIONAL PADRÃO**	
**Área:** ELSA-Brasil		**Página**: 1/3
**Assunto:** Técnica para mensuração de perímetro cefálico		**Vigência:** 10/05/2023

1. OBJETIVO

Estabelecer os critérios e as práticas para orientar as ações referentes à medição do perímetro cefálico.

2.RESPONSABILIDADES

2.2. Enfermeiro de pesquisa

3. DEFINIÇÕES

O perímetro cefálico é a medida da circunferência do crânio acima da cavidade supra-orbital e sobre a região occiptal na circunferência máxima, utilizando uma fita métrica inelástica e flexível, com precisão de 0,1 cm até 0,7cm.

4. DESCRIÇÃO DOS PROCEDIMENTOS

4.2.1. Perímetro cefálico

1. Higienizar as mãos;
2. Apresentar-se ao participante e explicar o procedimento a ser realizado;
3. Mantenha o participante sentado ou deitado;
4. Retirar qualquer objeto que possa alterar a medida, desde enfeites de cabelo a tranças ou outros penteados, com a autorização do participante;
5. Manter-se de frente para o lado esquerdo do participante e colocar o marco zero da fita na lateral da cabeça;
6. Posicionar a fita na região frontal da cabeça, acima das sobrancelhas e na parte posterior, na região occipital (osso localizado na base do crânio). Procurar sempre o maior diâmetro da cabeça, sem incluir as orelhas;
7. Observar se a fita está posicionada no mesmo nível em toda a cabeça, passando sobre a região mais saliente do occipital. Para isso, é necessária a ajuda de um assistente;
8. No momento do ajuste da fita, ela deve ser comprimida sobre o cabelo, quando houver, para se obter uma medida mais próxima da real circunferência cefálica. Fazer duas medidas e registrar a média entre elas;
9. Realizar a leitura.
10. Anotar o valor da medida na ficha de registro.

Figura 3 - Posicionamento da fita métrica para aferição de perímetro cefálico

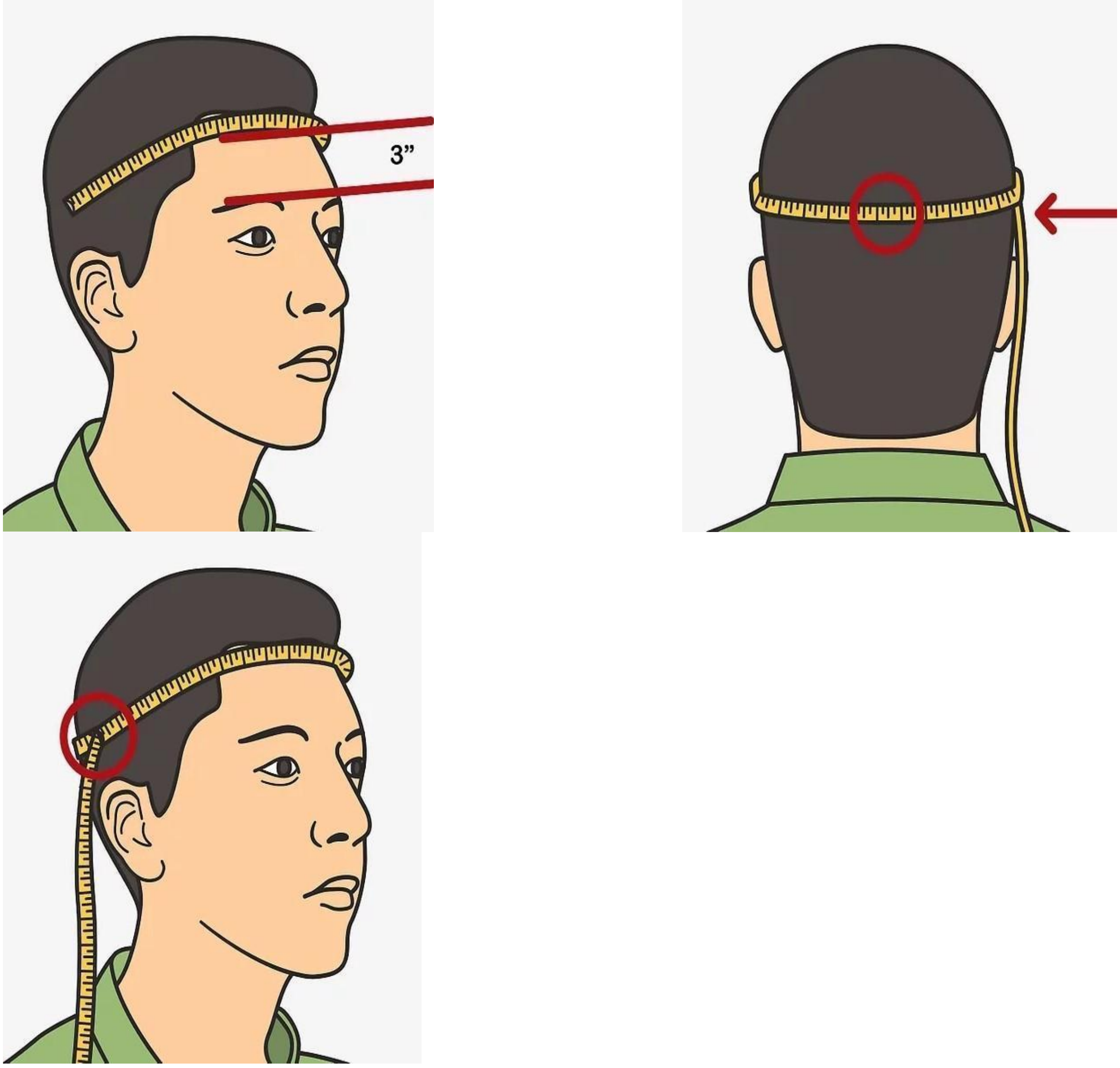

Fonte: wikiHow. Disponível em: https://pt.wikihow.com/Medir-a-Circunfer%C3%AAncia-da-Cabe%C3%A7a#aiinfo

- O medidor deverá circundar a fita acima da cavidade supra-orbital e sobre o occipital na circunferência máxima.
- Deve-se ter cuidado para que a fita esteja no mesmo nível em ambos os pontos supracitados.
- É preciso pressionar o suficiente para comprimir apenas o cabelo.
- A leitura deverá ser realizada no milímetro mais próximo.

Referências

1. Sampaio LR, Silva MCM, Oliveira TM, Ramos CI. Técnicas de medidas antropométricas. Avaliação nutricional. Salvador: EDUFBA; 2012. p. 89-101. Sala de aula collection. ISBN: 978-85-232-1874-4. Disponível em: https://books.scielo.org/id/ddxwv/pdf/sampaio-9788523218744-07.pdf

2. Barros DC, Felipe GC, Silva JP. Diagnóstico nutricional na atenção à saúde - Antropometria. Vigilância alimentar e nutricional para a saúde indígena. Rio de Janeiro: Editora FIOCRUZ; 2007. p. 33-74. Vol. 2. ISBN: 978-85-7541-589-4. Disponível em: https://books.scielo.org/id/c9fjf/pdf/barros-9788575415894-04.pdf

3. Macchiaverni LML, Barros Filho AA. Perímetro cefálico: por que medir sempre. Medicina (Ribeirão Preto) [Internet]. 30 de dezembro de 1998 [citado 10 de abril de 2023];31(4):595-609. Disponível em: https://www.revistas.usp.br/rmrp/article/view/7730

Elaborado por equipe do Estudo de Envelhecimento e Funcionamento Cerebral, ELSA- Brasil	10/05/23

Anexo 8. Obtenção de imagens no Home Images

	**Home Images**
**Área:** ELSA-Brasil	Página: 1/3
**Assunto:** Obtenção de imagens no *Home Images*	Vigência: 10/05/2023

1. OBJETIVO

Orientar sobre o passo a passo para obtenção das imagens dos exames de ressonância magnética realizados no 3T e 7T.

2. RESPONSABILIDADES

2.1 Enfermeiro de pesquisa, sanitarista

3. DEFINIÇÕES

3.1 A obtenção de imagens dos exames de ressonância magnética realizados tanto na máquina de 3T quanto na 7T são obtidos através do programa *home images*, na rede do Hospital das Clínicas, em computador disponibilizado pelo InRad (Instituto de Radiologia do Hospital das Clínicas FMUSP) e que tenha o acesso ao programa

4. DESCRIÇÃO DOS PROCEDIMENTOS PARA ARMAZENAMENTO DAS IMAGENS DE RM

4.1 Acesso ao Home Images 3T

4.1.1 Ligar o computador e colocar o login institucional e senha (@hc.fm.usp.br / @inrad.local).

4.1.2 No canto superior do Tela do computador, clique em “Go” -> “Connect to server” e selecione a opção: “Smb://nascluster.inrad.local/homeimages. A janela correta está descrito abaixo:

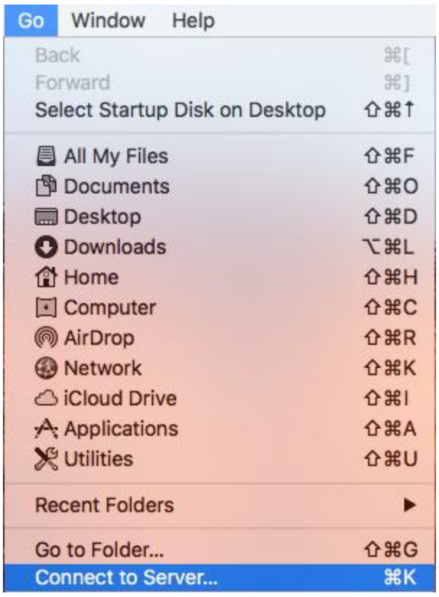

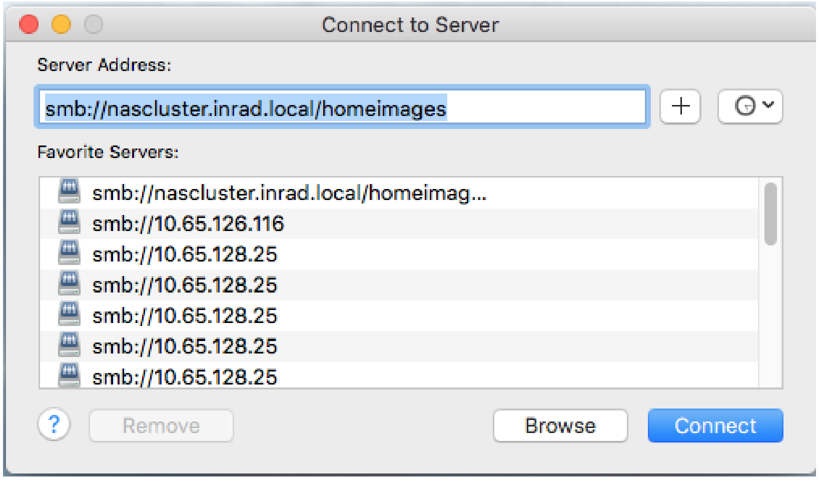

4.1.3 Após clicar em "continue", irá aparecer uma janela (como descrito na imagem abaixo). O acesso deverá ser feito com o e-mail institucional no servidor (@inrad.local) e a mesma senha do e-mail institucional.

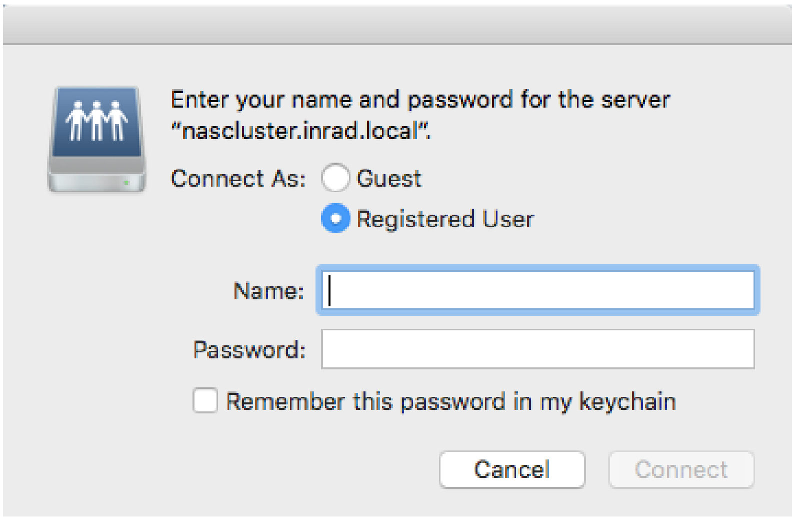

4.1.4 Logo após o acesso ao *Home Images*, a primeira maneira de capturar as imagens é filtrar por data de criação das pastas e verificar cada nome de pasta de participante com a seguinte sequência e informações:

- DataRealizaçãoExame_SiglasNomeParticipante_IDRecrutamento
  (Ex: 20230422_ADHJ_1042578).
  Observações: As datas são ao contrário; caso precise editar o nome da pasta avisar aos supervisores do estudo para comunicar a pessoa responsável pela correção (biomédico).

4.1.5 Conectar o HD Externo 3T na máquina

4.1.6 Selecionar as pastas que gostaria de fazer a transferência após conferir os dados no *Home Images*, clicar com o botão direito em cima da seleção e clicar em "Copy to x files".

- As pastas do 3T levam pelo menos 30 minutos para fazer a transferência de cada pasta. A demanda de pessoas usando o servidor também pode ocasionar o aumento do tempo dessa transferência.
- Antes da transferência das pastas, o programa irá sinalizar quantos arquivos e qual tempo estimado na barra que irá aparecer;

4.1.7 Clique com o botão direito no HD Externo e clique em "Past x files" e aguarde.

4.1.8 Uma segunda maneira de capturar as imagens após acessar o servidor, é através do terminal na máquina, ao digitar o seguinte código:
"cp -r homeimages/2024/182_2018_ELSA_INRAD/20240603_* ELSA\ Cer\ 3T/"

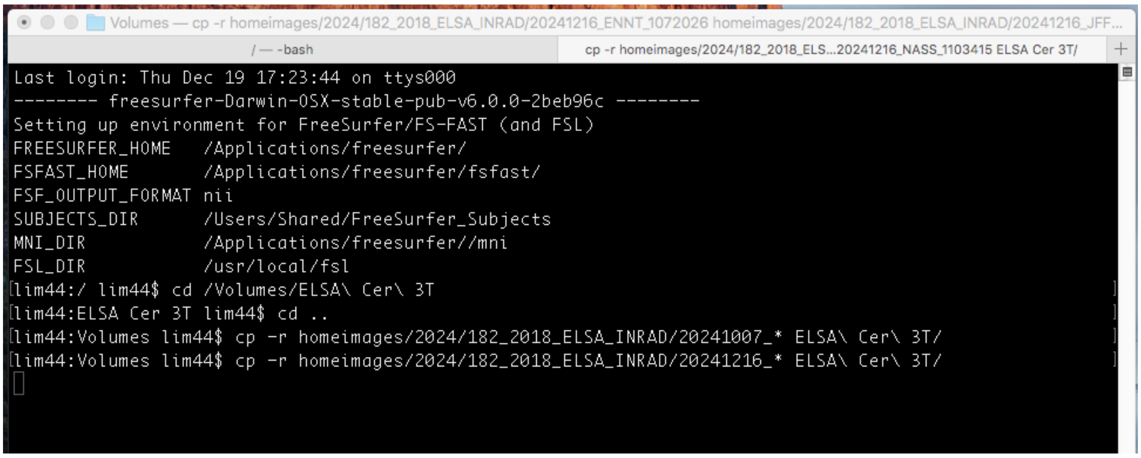

Por esse processo é possível identificar a pasta através do dia e mês de realização do exame.

4.1.9 Após aguardar a finalização da transferência, clique com o botão direito em cima da pasta da área de trabalho e clique em “Eject Home Images” para sair do seu login. Faça o mesmo para poder retirar o HD Externo.

4.2 Acesso ao Home Images 7T

4.2.1 Ligar o computador e acessar o home images

4.2.2 Acessar o programa visualizador de imagem, seguindo essas orientações:

No canto superior da tela do computador, clique em “Pacient id”, digite o código do estudo “Dro_0182”, “Retrieve” e selecione as opções do sistema virtual: PISA_DB, RM 7 TESLA. Filtre as informações clicando em “Any Date”, “CT”, “Retrieve to this computer”. A janela correta está como o anexo abaixo:

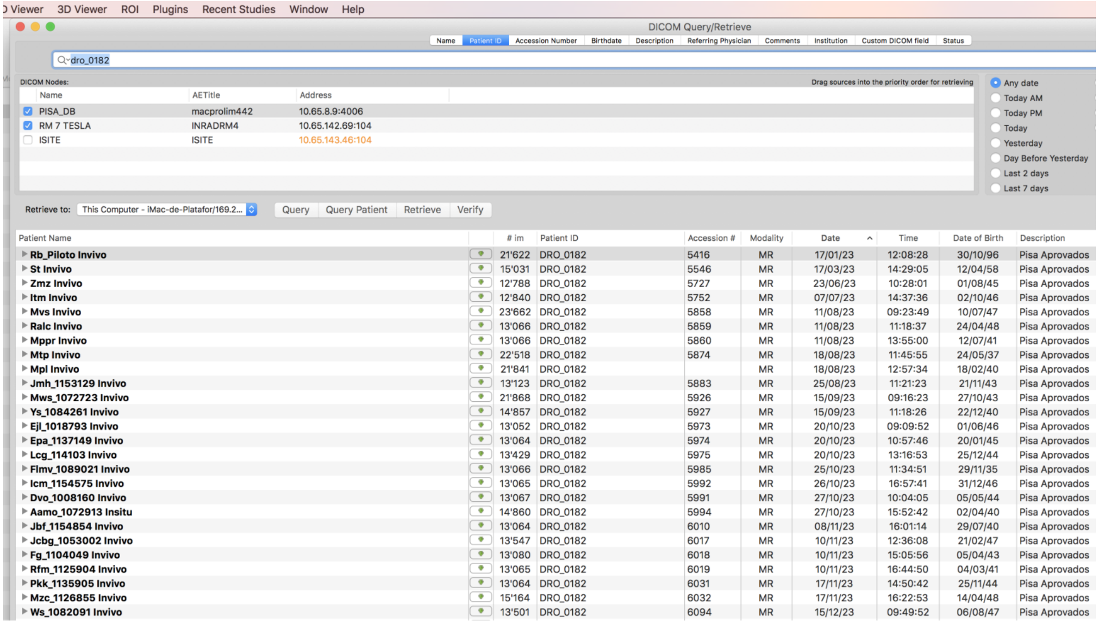

4.2.3 Após as imagens serem transferidas para o visualizador de imagem, realize a transferência dos arquivos para o HD Externo com as mesmas orientações acima. As pastas da 7 T levam pelo menos uma hora para fazer a transferência de cada pasta.

Essas informações originais foram descritas pelos próprios autores do estudo.

**Elaborado por equipe do Estudo de Envelhecimento e Funcionamento Cerebral, ELSA- Brasil**	10/05/23
