# Supplementary material for: Developing strategies and quality control in the Ageing and Brain Working Study in ELSA-Brasil
Source: Rev Saude Publica. 2026 Jan 12;59:e54. doi: 10.11606/s1518-8787.2025059006956 (PMC12795433; doi:10.11606/s1518-8787.2025059006956)
Supplement: Translation

# Translation: Etapas de desenvolvimento e controle de qualidade no Estudo de Envelhecimento e Funcionamento Cerebral no ELSA-Brasil

**Authors:** Alessandra Vanessa Lopes Quidim, Raphael Beuttler, Helena Mendes Eloi, Maria Concepción García Otaduy, José Gilvam Araújo Lima Júnior, Murillo Mariotto Teixeira, Carlos Eduardo de Lima Moraes Dardis, Khallil Taverna Chaim, Adriana Bastos Conforto, Claudia da Costa Leite, Carolina de Medeiros Rimkus, Claudia Kimie Suemoto, Angelita Gomes Souza, Itamar Souza Santos, Paulo Andrade Lotufo, Isabela Judith Martins Benseñor, Alessandra Carvalho Goulart

**Affiliations:** I Universidade de São Paulo. Hospital Universitário. Centro de Pesquisa Clínica e Epidemiológica. São Paulo, SP, Brasil; II Universidade de São Paulo. Hospital das Clínicas da Faculdade de Medicina. Instituto e Departamento de Radiologia, Laboratório de Investigação Médica em Radiologia 44. São Paulo, SP, Brasil; III Universidade de São Paulo. Faculdade de Medicina. São Paulo, SP, Brasil; IV Universidade de São Paulo. Faculdade de Saúde Pública. Departamento de Epidemiologia. São Paulo, SP, Brasil

**Keywords:** Gerenciamento de Dados, Gestão do Conhecimento para a Pesquisa em Saúde, Gestão de Ciência, Tecnologia e Inovação em Saúde, Manuais e Guias para a Gestão da Pesquisa

## Abstract

**OBJETIVO:**

Descrever todas as etapas de desenvolvimento e controle de qualidade no Estudo de Envelhecimento e Funcionamento Cerebral ligado ao Estudo Longitudinal de Saúde do Adulto (ELSA-Brasil), coorte prospectiva de 15.105 servidores públicos (35–74 anos) em seguimento desde 2008.

**MÉTODOS:**

Baseada na amostra de participantes vivos no início da seleção da amostra em setembro de 2022 (n = 4.566) do ELSA-Brasil (Centro Investigação São Paulo), foi calculada amostra representativa de 2.165 indivíduos segundo os seguintes critérios e divididos em duas subamostras: (1) Amostra de participantes < 70 anos na Onda 3 (2017–2019) randomizados de acordo com trajetória cognitiva da Onda 1 a 3, n = 1.670; (2) Amostra caso-controle *SuperAgers*, indivíduos com ≥ 70 anos na Onda 3, baseado na memória tardia da Onda 3, n = 495 participantes (171 casos, 324 controles). Os processos implementados para assegurar o controle de qualidade das informações antes da coleta de dados foram: a seleção dos instrumentos de coleta de dados, treinamento e certificação das equipes, elaboração de manual de definições e procedimentos operacionais padronizados, estudos pilotos, fluxo de coleta de dados, harmonização dos protocolos de imagem e criação de dispositivo antirruído. Dada a abrangência do estudo, a natureza multicêntrica e a diversidade de medidas envolvidas, este projeto demandou a implementação de protocolos eficazes de garantia e controle de qualidade, assegurando padronização, consistência metodológica e integridade dos dados em todas as etapas.

**RESULTADOS:**

Após a implementação dos processos de garantia e controle de qualidade, foram conduzidas as etapas finais relacionadas à coleta de dados clínicos e de imagem, gerenciamento e monitoramento contínuo das atividades de campo, com elaboração de relatórios semanais e acompanhamento estatístico da amostra.

**CONCLUSÕES:**

A adoção de etapas sistematizadas de desenvolvimento e controle de qualidade foi fundamental para garantir a produção e confiabilidade de informações de estudos de neuroimagem cerebral.

## INTRODUÇÃO

Estudos epidemiológicos caracterizados pelo uso de métodos padronizados e delineados contemplando as especificidades de uma população são essenciais para subsidiar dados confiáveis. A qualidade dos dados produzidos em estudos de coorte, que se caracterizam por acompanhamento de longo prazo, grande detalhamento e checagem de informações coletadas demandam um controle de qualidade rigoroso para garantir a excelência dos resultados obtidos^1^.

Nesse contexto, o Estudo Longitudinal de Saúde do Adulto (ELSA-Brasil) coorte multicêntrica, que se propõe a investigar a incidência e os fatores de risco para doenças crônicas, em particular, as doenças cardiovasculares e o diabetes^2,3^em andamento em seis centros de excelência em pesquisa e ensino, de 15.105 funcionários públicos de ambos os sexos (35 a 74 anos), se destaca pela garantia de um controle de qualidade bastante rigoroso desde sua concepção (linha de base: 2008–2010)^4^.

Após 14 anos do início do estudo principal, com o envelhecimento da coorte foi proposto um estudo ancilar para avaliar o envelhecimento cerebral pela captação de neuroimagem de alta resolução [ressonância magnética (RM) 3 (3T) e 7 (7T) Tesla]. O Estudo de Envelhecimento e Funcionamento Cerebral (*Ageing and Brain Working Study*) ligado ao ELSA-Brasil tem como principal objetivo avaliar os determinantes do processo de envelhecimento cerebral, intimamente relacionados à trajetória cognitiva e de saúde mental, que podem estar associados a agravos crônicos e fatores de risco não transmissíveis, como os de origem cardiovascular e hábitos de vida. Além disso, esse estudo se propõe a investigar o envelhecimento bem-sucedido com o estudo dos *SuperAgers* (Super Idosos): indivíduos com mais de 80 anos e desempenho de memória semelhante a pessoas saudáveis duas a três décadas mais jovens^5^. Considerando-se a multidimensionalidade das variáveis de exposição coletadas desde o início do estudo principal, a diversidade da amostra em relação às características sociodemográficas, tais como raça, idade, sexo e nível educacional, diferentemente de outras coortes de países desenvolvidos que avaliam o envelhecimento^6^, incluindo estudos de SuperAgers^9^, o ELSA-Brasil avança frente a essa nova realidade de maneira única e inovadora para um conhecimento mais aprofundado na área. Portanto, por meio da elaboração, implementação de protocolos eficazes de garantia e controle de qualidade, padronização e consistência metodológica, o ELSA-Brasil garante uma maior integridade dos dados em todas as etapas do estudo a longo prazo.

O objetivo principal deste artigo é descrever as etapas de desenvolvimento e controle de qualidade e as medidas adotadas para assegurar o controle de qualidade das informações geradas pelo Estudo de Envelhecimento e Funcionamento Cerebral em um dos Centros de Investigação ELSA-Brasil, em São Paulo.

## MÉTODOS

### População e Desenho do Estudo

Trata-se de um estudo de coorte prospectiva para avaliar o processo de envelhecimento cerebral no ELSA-Brasil.

Brevemente, ELSA-Brasil é uma coorte multicêntrica, composta por 15.105 servidores públicos, com idades entre 35 e 74 anos na linha de base, recrutados entre 2008 e 2010, que tem como principal objetivo investigar a incidência e os fatores de risco para doenças crônicas, em particular, as doenças cardiovasculares e o diabetes^2,3^. Assim como proposto nesse estudo ancilar, o ELSA-Brasil como coorte pioneira brasileira de longo prazo também adotouestratégias para assegurar o controle de qualidade dos dados coletados desde a linha de base^4^.

Para o presente estudo foi considerada uma amostra representativa de participantes vivos (n = 4.566) do Centro Investigação São Paulo do ELSA-Brasil^2^. Calculou-se uma amostra de 2.165 indivíduos baseados nos seguintes critérios e divididos em duas subamostras: (1) Amostra de participantes com menos de 70 anos na Onda 3 (2017–2019) perfazendo um total de 1.670, randomizada com base na trajetória cognitiva da Onda 1 a 3 calculada pelo *z-score* total predito por *regression based norms* pelos dados da bateria cognitiva (escores de memória + fluência + teste de trilhas) de acordo com idade, escolaridade e sexo e (2) Amostra para o estudo de caso-controle *SuperAgers*, indivíduos com 70 anos ou mais que apresentavam pontuações de memória tardia iguais ou superiores à mediana das pontuações dos indivíduos com idades compreendidas entre os 46 e os 54 anos na Onda 3, em comparação aos seus pareados por sexo e idade, perfazendo um total de 495 participantes (casos = 171, controles = 324).

Nesse estudo de envelhecimento foi desenvolvido um protocolo de pesquisa padronizado para coleta de dados clínicos com foco em cognição, saúde mental e de neuroimagem de alta resolução (RM 3 e 7 Tesla) para o estudo aprofundado do processo de envelhecimento cerebral estrutural e funcional, levando-se em consideração a harmonização dos dados de imagem com protocolos internacionalmente utilizados em outras coortes que incluíram dados de neuroimagem e que, à semelhança do ELSA-Brasil, também se dedicam ao estudo de determinantes do envelhecimento cerebral^2,3,6^. Os processos implementados para assegurar o controle de qualidade das informações, antes de iniciar a coleta de dados, foram: a seleção dos instrumentos de coleta de dados, treinamento e certificação das equipes, elaboração de manual de definições e procedimentos operacionais padronizados (POPs), estudos-piloto, fluxo de coleta de dados, harmonização dos protocolos de imagem e criação de dispositivo antirruído (Figura 1). Todos esses processos foram detalhados ao longo do texto.

**Figura 1:** Etapas de desenvolvimento e controle de qualidade do ELSA-Brasil.

O Quadro 1 apresenta a logística e os processos desenvolvidos pelo estudo.

**Quadro 1:** Logística e procedimentos para elaboração do estudo de neuroimagem cerebral no ELSA-Brasil (Estudo de Envelhecimento e Funcionamento Cerebral).

.
Desenvolvimento do projeto de pesquisa
Definição e pré-teste do protocolo de imagem
Seleção e Pré-teste dos questionários e escalas validados de rastreamento com foco em cognição, saúde mental, funcionalidade e fragilidade
Manuais de definições de termos médicos e procedimentos operacionais padronizados
Treinamento e certificação da equipe de coleta de dados e agendamento
Pilotos de exames de imagem na RM 3T e 7T para verificar consistência, reprodutibilidade em tempo hábil tolerável pelo participante
Pilotos para verificação de fluxo de coleta de dados em campo, incluindo tempo total por participante (leitura e assinatura de documentos, preenchimento dos questionários e troca de vestimenta)
Coleta de dados preliminares
Adaptação dos instrumentos de pesquisa
Desenvolvimento de dispositivo para fixação e antirruído para realização da RM
Protocolo para armazenamento de imagens em um HD externo (back-up), além do ambiente virtual
Protocolo de aquisição e arquivamento de dados clínicos
Gerência de dados e relatórios periódicos

RM: ressonância magnética; HD: *hard disk.*

### Desenvolvimento do Projeto de Pesquisa

O processo de criação do protocolo de pesquisa padronizado para coleta de dados de neuroimagem de alta resolução e avaliação de envelhecimento cerebral estrutural e funcional levou em consideração os objetivos do estudo com foco no envelhecimento, delineamento prospectivo com seguimento até dois anos após a captação da neuroimagem, características da população, incluindo a avaliação de participantes acima de 70 anos, o tamanho da amostra e escolha inicial do protocolo de imagem e instrumentos de coleta de dados a serem aplicados para avaliação multidimensional do envelhecimento cerebral em homens e mulheres.

A equipe de pesquisa ponderou aspectos logísticos, operacionais e financeiros ao longo de todo o processo, considerando a opinião, o entendimento e a aceitabilidade dos participantes para os ajustes realizados no protocolo original, tanto na fase piloto quanto durante a execução do estudo. Uma equipe de pesquisadores clínicos (epidemiologistas, neurologistas, geriatras e psiquiatras), neurorradiologistas e físicos médicos se organizaram em comitês específicos para definir o protocolo de imagens e selecionar os dados clínicos coletados.

### Protocolo de Imagem

Para a padronização do protocolo foi considerada a obtenção de uma sequência de imagens com duração aproximada de uma hora, tanto para a RM 3T como para 7T. Posteriormente, realizou-se a harmonização com protocolos internacionais utilizados em outras coortes que avaliaram o envelhecimento cerebral, seguindo parâmetros comparáveis à coorte *European Prevention of Alzheimer’s Dementia Longitudinal Cohort Study (*EPAD*)* ^6^. As sequências multiplanares ponderadas estão descritas no Quadro 2. O protocolo foi realizado sem o uso de contraste paramagnético (gadolínio), exceto se identificada imagem suspeita de lesão ativa (ex.: tumor, meningioma). Nesse caso, realizou-se nova convocação para repetir o exame com uso de contraste.

**Quadro 2:** Principais sequências com respectivas características das imagens adquiridas na ressonância magnética (RM 3T e 7T) no Estudo de Envelhecimento e Funcionamento Cerebral (ELSA-Brasil).

Características principais	Ressonância magnética 3T Intera Achieva, PHILIPS Healthcare (Best, Holanda) com bobina de cabeça de 32 canais	Ressonância magnética 7T Magnetom, Siemens Healthineers (Erlangen, Alemanha) com bobina de cabeça de 32 canais
Imagem anatômica ponderada em T1, útil para a segmentação das estruturas encefálicas, avaliação de atrofia, alargamento de espaços perivasculares, avaliação de lacunas e microinfartos.	**Sagital 3D T1- weighted fast field echo (FFE)** Resolução isotrópica de 1 mm	**Sagital 3D MP2RAGE** Resolução isotrópica de 0,75 mm
Imagem ponderada em T2 com supressão do líquido cefalorraquidiano, útil para visualização de lesões hiperintensas, que podem estar associadas a áreas de gliose, doença (micro)vascular intracraniana, infartos sequelares, lesões desmielinizantes etc.	**Sagital 3D fluid attenuated inversion recovery (FLAIR)** Resolução isotrópica de 1 mm	**Sagittal 3D fluid attenuated inversion recovery (FLAIR)** Resolução isotrópica de 0,75 mm
Imagem ponderada em T2, útil para visualização de patologias com maior conteúdo de água, como edema, cistos ou líquido cefalorraquidiano. Detecta lesões hiperintensas semelhantes às detectadas em FLAIR, com melhor visualização de lacunas, alargamento de espaços perivasculares e microinfartos corticais, principalmente quando analisados em conjunto com as demais sequências estruturais.	**Axial 2D T2-weighted fast spin-echo (FSE**) Resolução de 0,9 mm no plano e 3 mm de espessura de fatia	**Sagital 3D T2 Space** Resolução isotrópica de 0,7 mm
Imagem ponderada em susceptibilidade magnética, útil para visualização dos seguintes fatores: 1) Depósito de ferro na substância cinzenta profunda e em outros compartimentos cerebrais: associado ao envelhecimento e várias doenças neurodegenerativas, 2) Depósito de hemossiderina: sangramento crônico ou antigo, focos de microhemorragia associados à angiopatia amiloide e/ou doença vascular intracraniana; 3) Depósito cálcio: neurocisticercose, granulomas calcificados e outras calcificações anômalas.	**3D Susceptibility-weighted image (SWI)** Resolução de 0,5 mm no plano e 3 mm de espessura de fatia	**3D Susceptibility-weighted image (SWI)** Resolução de 0,21 mm no plano e 1,5 mm de espessura de fatia Axial **3D FFE multi-gradient echo**: resolução isotrópica de 0,7 mm. Sequência realizada para obtenção dos mapas quantitativos de susceptibilidade (QSM -*quantitative susceptibility mapping*), útil na avaliação complementar do SWI, para a avaliação quantitativa dos depósitos e metabolismo do ferro.
Imagem ponderada em difusão, útil para detecção de infarto agudo e para análise da microestrutura cerebral, tractografia e conectividade estrutural.	**Axial 2D Diffusion-Weighted (DWI) SE-EPI** Resolução isotrópica de 2 mm, com dois valores de b: 0 e 1000 s/mm^2^, 48 direções de gradiente	**Axial 2D Diffusion-weighted (DWI) SE-EPI** Resolução isotrópica de 2 mm, com três valores de b: 0,1000 e 2000 s/mm^2^, 30 direções de gradiente
Imagem dinâmica ponderada no contraste *blood oxygenation level dependent* (BOLD) no estado de repouso, útil para avaliação da atividade cerebral e conectividade funcional.	**Axial 2D Resting state-fMRI GRE-EPI** Resolução de 3,3mm	**Axial 2D Resting state-fMRI GRE-EPI** Resolução de 2,2mm
Imagem dinâmica de *arterial spin labeling* (ASL), útil para avaliação da perfusão cerebral sem necessidade da injeção de contraste.	**Axial 3D GRASE com ASL** Resolução de 3,75 mm no plano e 6 mm de espessura de fatia	

RM: ressonância magnética.

A primeira avaliação foi uma análise visual das imagens obtidas, a fim de avaliar a qualidade da imagem, presença de artefatos, quantificação de lesões de substância branca (lesões de hiperintensidade) e avaliação de lesões estruturais cerebrais (Material suplementar: Anexo 1)^a^.

As imagens foram avaliadas por dois neurorradiologistas com, no mínimo, 10 anos de experiência na área. Cada exame foi interpretado por apenas um neurorradiologista, que efetuou a leitura dos achados qualitativos, descritos no Anexo 1. Caso houvesse discordância, um consenso era feito entre os especialistas. Achados relevantes, com potencial impacto no acompanhamento clínico do participante, como tumores intracranianos, hemorragias, infartos ou outras lesões estruturais relevantes foram descritos em um laudo sumarizado e entregue aos respectivos participantes. Os demais achados qualitativos foram armazenados no banco do estudo.

### Seleção dos Questionários de Pesquisa

Os instrumentos aplicados visaram o rastreamento de sintomas relacionados ao processo de envelhecimento como o *status* cognitivo (entrevista telefônica para avaliação do estado cognitivo - versão modificada (*modified telephone interview for cognitive status*,TICS-M))^15^, avaliação funcional (questionário de atividades funcionais de Pfeffer^16^ e questionário de fragilidade-frail)^17^ e saúde mental pela aplicação das seguintes escalas: escala de dimensões de raiva (*dimension of anger reactions*, DAR-5)^18^, escala de afeto positivo e negativo (*positive and negative affect schedule*, PANAS)^19^, escala de ansiedade-traço (IDATE)^20^, questionário de depressão (*patient health questionnaire*, PHQ-9)^13^, escala de depressão geriátrica (GDS-15)^21^ e escala de distresse (K-6)^22^. Além disso, instrumentos sobre hábitos de vida, incluindo aspectos da dieta^23^, como consumo de café, alimentos processados^20^ e álcool, tabagismo, atividade física e sono^23,24^. Os instrumentos utilizados no estudo foram validados no Brasil com reprodutibilidade e confiabilidade bem estabelecidas na população idosa^25^.

### Manual de Definições e Procedimentos Operacionais Padronizados

O manual de definições incluiu as doenças clínicas e as principais urgências e emergências mais prevalentes na população, com linguagem acessível e padronizada para letramento de toda equipe de pesquisa.

Os POPs elaborados constituíram uma compilação detalhada de todas as atividades realizadas, com descrições claras e minuciosas relacionadas à aferição de sinais vitais, mensuração de perímetro encefálico e orientações para acionamento do código azul do Instituto Central do Hospital das Clínicas (ICHC)/Instituto de Radiologia (InRad), incluindo uma equipe de resposta rápida multidisciplinar, treinada para atender pacientes em parada cardiorrespiratória “Código Azul” ou intercorrência clínica aguda e grave “Código Amarelo” em áreas não críticas do ICHC e seu entorno (Material suplementar: Anexos 2 a 8)^b^.

### Recrutamento da Equipe de Pesquisa

O estudo teve início com a identificação e recrutamento de profissionais qualificados para compor a equipe do estudo. Após a seleção inicial, diversas reuniões foram realizadas com o pesquisador principal e os coordenadores para discutir as necessidades operacionais do projeto. Simultaneamente, começou a aquisição de materiais essenciais, tais como computadores, aparelhos de telefonia, *tablets*, dispositivos externos de armazenamento de dados clínicos e de imagem, definição dos kits de lanches não perecíveis, protetores auriculares para uso durante o exame, cadeiras com apoio para destros e canhotos para preenchimento dos questionários, materiais de papelaria e produtos de desinfecção hospitalar.

### Treinamento e Certificação de Segurança para Realização da RM

Foi realizado um treinamento específico, antes do início da coleta de dados, abordando os cuidados necessários para o posicionamento adequado dos participantes, as instruções relacionadas à sua segurança durante a realização dos exames de RM 3T e 7T, e treinamento para aplicação do questionário de segurança em ambiente de RM.

Para garantir a segurança da equipe de campo durante a condução do estudo na área dos exames de RM, exigiu-se leitura e assinatura do termo de boas práticas para pesquisa no Laboratório de Investigação Médica em RM em Neurorradiologia (LIM44) e um curso de certificação *online* sobre Segurança em RM pela equipe responsável pelo setor de neuroimagem do InRad-ICHC, FMUSP.

### Treinamento e Certificação para Coleta de Dados Clínicos

Um treinamento envolvendo a equipe de pesquisa responsável pela coleta dos dados em campo foi realizado: (1) enfermeira gerente de campo responsável pelo acolhimento do participante, orientação sobre a coleta de dados clínicos e de imagem em campo, (2) sanitarista responsável pelo gerenciamento, seleção, e arquivamento das imagens adquiridas e (3) dois enfermeiros designados para coleta de dados, aplicação do termo de consentimento livre e esclarecido (TCLE) e orientação para preenchimento correto dos questionários/escalas via *tablet*.

O treinamento e certificação dos instrumentos clínicos, com carga horária total de 9 horas, foi realizado pela equipe do ELSA-Brasil responsável pelo controle de qualidade dos questionários do Centro São Paulo, com foco na aplicação de escalas e questionários.

Além do treinamento para aplicação presencial, os entrevistadores também foram certificados para aplicação dos mesmos questionários via telefone nos anos 1 e 2 após a aquisição da neuroimagem. A equipe também foi treinada para realização de mensuração de medidas antropométricas (peso, altura), perímetro cefálico, medida casual da pressão-arterial, baseadas em técnicas consolidadas^3,30,31^.

A dicção – incluindo a tonalidade da voz e as pausas – e o tempo de aplicação e orientação dos questionários e escalas também foram padronizados^3^. Esses elementos foram considerados requisitos centrais para garantir que as perguntas fossem interpretadas de maneira uniforme ao longo das coletas. O treinamento da dicção mostrou-se fundamental para assegurar precisão, clareza e comunicação eficaz, contribuindo para a compreensão adequada das perguntas e para a qualidade das respostas. A tonalidade controlada, em tom neutro e bem modulada, favoreceu a empatia, criando um ambiente de confiança – especialmente importante em contextos sensíveis, como os que envolvem saúde mental.

Além disso, a leitura de palavras específicas com ênfase e a realização de pausas estratégicas possibilitaram melhor processamento e compreensão das questões pelos participantes, o que contribuiu para a validade das respostas obtidas. A conscientização da equipe sobre a importância de respeitar os intervalos de tempo recomendados para a aplicação dos instrumentos, bem como a manutenção de uma postura e dicção neutras – sem influenciar as respostas – reforçou o rigor metodológico do estudo^3^.

Assim, investir nesse treinamento foi fundamental para obter dados de alta qualidade e confiabilidade.

Em média, a aplicação do protocolo presencial levou em torno de 90 minutos (questionários, escalas e TCLE). Um grande desafio foi o treinamento dos entrevistadores para orientar a autoaplicação de todos os instrumentos e escalas, exceto a TICS-M, em indivíduos mais idosos (idade média de 67 anos, variando de 47 a 90 anos na linha de base), particularmente.

### Treinamento para o Agendamento dos Casos

O agendamento dos participantes foi responsabilidade de uma equipe especializada, composta por um enfermeiro e um sanitarista, que também participaram de simulações práticas para o convite e a aplicação de questionários por telefone, utilizando roteiros padronizados preparados pela supervisora do projeto e pelos coordenadores de campo. Foi realizado treinamento adicional para o preenchimento da documentação com dados sensíveis dos participantes, solicitação de exames, envio de e-mails padronizados ao InRad e aos participantes, incluindo mensagens de lembrete enviadas 48 horas antes do exame, com o intuito de reduzir ao máximo faltas ou desistências.

Dois treinamentos com certificação e recertificação foram realizados em 2023, antes do início do estudo, com foco nos aspectos do agendamento por telefone da RM e nas especificidades envolvidas no processo de obtenção de uma sequência de imagens de qualidade. Entre essas orientações, desatacaram-se a recomendação de manter uma alimentação leve (sem necessidade de jejum prévio) e a importância de permanecer com a cabeça imóvel durante o tempo em que o participante estiver no interior do equipamento.

### Pilotos do Estudo

A equipe do estudo realizou exames piloto nos equipamentos de RM com o objetivo de otimizar a logística e antecipar possíveis barreiras que poderiam dificultar a coleta de dados clínicos e de imagem. O principal objetivo foi estabelecer um protocolo de imagem consistente, reprodutível e viável dentro de um tempo tolerável para os participantes do estudo.

Ao final do piloto, definiu-se que o tempo de cada exame na máquina de RM 3T seria de aproximadamente uma hora. As etapas de recepção, assinatura do TCLE e aplicação de questionários totalizaram cerca de 3 horas e 30 minutos, na ausência de intercorrências.

No dia do exame de RM 7T, aplicaram-se dois questionários breves: um de segurança, antes do procedimento, e outro após o exame, para avaliar desconfortos ou sintomas relatados durante a realização da imagem. O tempo total estimado para essa etapa foi, em média, de duas horas, também na ausência de intercorrências.

### Coleta de Dados Preliminares e Adaptação dos Instrumentos

Após a definição dos fluxos de atendimento, os instrumentos selecionados tiveram sua compreensão avaliada pela equipe do estudo após a inclusão dos 20 primeiros participantes. Com base nessa etapa, pequenas modificações e adaptações foram realizadas, sem comprometer a validade e o conteúdo dos instrumentos. A equipe executora foi devidamente treinada para o uso correto dos instrumentos, garantindo consistência na coleta dos dados.

Esse processo assegurou que os instrumentos de pesquisa fossem adequados, validados e confiáveis para a população-alvo e compatíveis com os objetivos do estudo.

### Desenvolvimento de Dispositivo para Estabilização da Cabeça e Antirruído para Realização da RM

O estudo-piloto, realizado com cinco participantes voluntários (entre 40 e 65 anos), identificou que a principal queixa durante os exames de RM foi o ruído gerado pela máquina, especialmente nos exames de RM 7T, mesmo com o uso de protetores auditivos padrão. Essa queixa foi preocupante, pois poderia levar a movimentos indesejados da cabeça, comprometendo a qualidade da imagem obtida.

Em resposta a esse problema, a equipe de pesquisa – composta por um físico médico e um tecnólogo especialista em biomecânica e tecnologia assistiva – iniciou, em 2023, o desenvolvimento de um protótipo estabilizador de cabeça e redutor de ruído, inspirado em modelos já comercializados na Europa e nos Estados Unidos, mas que no Brasil só estão disponíveis por meio de importação de alto custo. O novo protótipo proporciona uma estabilização confortável da cabeça, assegurando melhor qualidade de imagem durante os exames.

A equipe avaliou o material original do dispositivo comercializado internacionalmente e, a partir disso, iniciou a confecção do protótipo, que consiste em duas bolsas de PVC para cada orelha, conectadas a duas cânulas de borracha e três válvulas em resina. Essas válvulas foram produzidas por impressão 3D e permitem a insuflação das bolsas, que são vedadas por meio de uma seladora. O sistema proporciona estabilização da cabeça e redução do ruído, melhorando significativamente a sensação acústica durante o exame.

O protótipo foi testado com participantes da pesquisa, e todos os materiais utilizados foram considerados seguros para uso em ambientes com campo magnético. A Figura 2 apresenta a seladora desenvolvida para vedação das bolsas de PVC, bem como os demais materiais usados (conectores de borracha e válvulas de resina). Os resultados desses testes puderam contribuir significativamente para o aprimoramento da experiência dos participantes e para a melhoria da qualidade das imagens obtidas em exames de RM.

**Figura 2:** Protótipo do protetor antirruído produzido pela equipe de pesquisa durante o estudo ELSA-Brasil.

### Protocolo de Armazenamento de Dados de Imagem

Desenvolveu-se também um protocolo para o armazenamento das imagens em um disco rígido externo, além das plataformas em nuvem do InRad e do PISA. Após a realização dos exames, as imagens foram extraídas dos sistemas virtuais do InRad (RM 3T) e do PISA (RM 7T) e transferidas para armazenamento externo em mídias ópticas – *compact disc/digital versatile disc* (CD/DVD) – devidamente identificadas com o nome do participante, ID_ELSA, data e hora do exame, data de nascimento, RGHC, número de identificação no sistema virtual do InRad, nome do protocolo e tipo de exame realizado.

Em conformidade com a Lei Geral de Proteção de Dados Pessoais, todo o acesso às imagens dos participantes foi protegido por senha, restrito exclusivamente à equipe operacional e aos pesquisadores responsáveis.

## RESULTADOS

Após a implementação dos processos de garantia e controle de qualidade prévios à coleta de dados, foram conduzidas as etapas finais relacionadas à coleta de dados clínicos, ao gerenciamento e ao monitoramento contínuo das atividades de campo, com a elaboração de relatórios semanais e acompanhamento estatístico da amostra.

A coleta de dados clínicos foi realizada por meio de formulários digitais (Google Forms^®^), autopreenchidos pelos participantes em tablets acessados com senha individual, fornecida pela equipe de pesquisa, sob supervisão em tempo real dos entrevistadores do ELSA-Brasil. Ao término do preenchimento, os dados foram automaticamente armazenados em planilhas estruturadas, gerenciadas pelos entrevistadores e supervisores de campo. Essa estratégia assegurou a integridade, a confidencialidade e a rastreabilidade das informações, otimizando seu uso nas etapas subsequentes da pesquisa. Os dados foram mantidos em ambiente seguro (Google Drive^®^), com acesso restrito à equipe de campo e aos pesquisadores principais – estes com permissão apenas para leitura. Eventuais inconsistências nos questionários foram verificadas e corrigidas exclusivamente pela equipe de gerenciamento de dados com supervisão diária realizada por dois coordenadores. O monitoramento da equipe do Estudo de Envelhecimento e Funcionamento Cerebral do ELSA-Brasil foi intensivo e estruturado, envolvendo uma supervisão diária por dois coordenadores. Esse acompanhamento abrangeu todo o processo de coleta de dados, incluindo o gerenciamento da documentação, previsão e provisão de recursos humanos, alimentícios e materiais, monitoramento dos bancos de dados e das estatísticas, controle de laudos, bem como o gerenciamento e armazenamento das imagens. As observações periódicas tinham como objetivo identificar a necessidade de ajustes no fluxograma de atendimento, desvios de protocolo e aspectos da qualidade da coleta de dados. Utilizaram-se fluxogramas de atendimento, *checklists* e *scripts* padronizados para a aplicação do TCLE e dos questionários.

Os entrevistadores realizaram a verificação das respostas preenchidas pelos participantes durante a aplicação dos questionários, a fim de identificar inconsistências e/ou erros, possibilitando a correção imediata com a presença do participante. Relatórios semanais e estatísticas da amostra foram produzidos pelos supervisores de campo, assegurando o cumprimento das metas de recrutamento e o controle da adesão dos participantes.

Por fim, criou-se um mecanismo de feedback para os participantes, com o objetivo de avaliar a qualidade do atendimento e a receptividade da equipe. As opiniões, sugestões e críticas foram recebidas por meio de um aplicativo de mensagens.

## DISCUSSÃO

A quantificação do envelhecimento biológico, especialmente no contexto do envelhecimento cerebral, é essencial para a compreensão dos mecanismos subjacentes ao declínio cognitivo e para a identificação de fatores de risco e proteção ao cérebro no envelhecimento ao longo do tempo.

No contexto brasileiro, o ELSA-Brasil destaca-se pela sua metodologia robusta, que inclui estratégias abrangentes de garantia de qualidade, como calibração constante dos equipamentos, auditorias sistemáticas e treinamento contínuo das equipes envolvidas na coleta e análise dos dados^2,3^. A integração recente de avaliações neuropsicológicas e de neuroimagem amplia o potencial do ELSA-Brasil para investigar não apenas o envelhecimento típico (declínio cognitivo), mas também o envelhecimento dos *SuperAgers*, que apresentam desempenho cognitivo igual ou superior ao de pessoas muito mais jovens, especialmente em memória.

De maneira complementar, o Rotterdam Study^6^ é uma referência consolidada no cenário internacional, com mais de três décadas de acompanhamento rigoroso e padronizado de uma coorte populacional. A manutenção da consistência metodológica ao longo do tempo, por meio de protocolos clínicos e laboratoriais uniformes, bem como sistemas centralizados de controle de dados, tem permitido a identificação de biomarcadores precoces e trajetórias de envelhecimento saudável e patológico.

Estudos longitudinais de grande porte, como o ELSA-Brasil^2,3^e o Rotterdam Study^6^, foram fundamentais nesse cenário, pois possibilitaram a análise de trajetórias diferenciadas de envelhecimento a partir da coleta sistemática de dados clínicos, cognitivos e de neuroimagem. A confiabilidade dessas análises depende diretamente da implementação rigorosa de protocolos padronizados e do controle de qualidade, assegurando a validade e a comparabilidade dos resultados entre diferentes fases do estudo e entre diferentes populações.

Outros estudos realizados em países desenvolvidos como Estados Unidos, Espanha e Austrália que avaliaram amostras de *SuperAgers* compartilharam a preocupação com a preservação cognitiva no envelhecimento, com seleção cuidadosa da amostra e um controle rigoroso da qualidade metodológica e dos dados, garantindo que os achados sejam confiáveis e úteis para entender os fatores que protegem o cérebro no envelhecimento saudável^9^.

Dessa forma, a integração dos achados desses diferentes estudos^2,3,6^reforça a importância do controle de qualidade e da padronização na mensuração do envelhecimento biológico, contribuindo para o desenvolvimento de estratégias eficazes a fim de promover o envelhecimento cerebral saudável e prevenir o declínio cognitivo em larga escala.

## CONCLUSÃO

A implementação de etapas sistematizadas de desenvolvimento e controle de qualidade foi essencial para assegurar a produção e a confiabilidade dos dados em estudos de neuroimagem cerebral de alta resolução (3T e 7T), voltados à investigação do envelhecimento funcional e estrutural, considerando a complexidade metodológica e a sensibilidade intrínseca das informações obtidas no Estudo de Envelhecimento e Funcionamento Cerebral do ELSA-Brasil.

## Footnotes

a Material suplementar disponível em: https://figshare.com/s/da0e932b6f1f0b36fae5

b Material suplementar disponível em: https://figshare.com/s/da0e932b6f1f0b36fae5
